# Supplementary material for: A reporter system coupled with high-throughput sequencing unveils key bacterial transcription and translation determinants
Source: Nat Commun. 2017 Aug 28;8:368. doi: 10.1038/s41467-017-00239-7 (PMC5573727; doi:10.1038/s41467-017-00239-7)
Supplement: Supplementary file 1 — Supplementary Information [file 41467_2017_239_MOESM1_ESM.pdf]

File name: Supplementary Information
Description: Supplementary figures, supplementary tables, supplementary notes and supplementary references.

File name: Supplementary Data 1
Description: Oligonucleotides used in this study

File name: Supplementary Data 2
Description: Top 3 quantification of proteins from LC-MSMS data

File name: Supplementary Data 3
Description: Enriched sequences in the -17 to -13 positions of productive promoters

File name: Supplementary Data 4
Description: Number of transcripts with different transcription start sites

File name: Supplementary Data 5
Description: Pearson correlation between mRNA folding energy and DAMRatio

File name: Supplementary Data 6
Description: 5'-UTR translation strength prediction power from individual features

File name: Peer review file
Description:

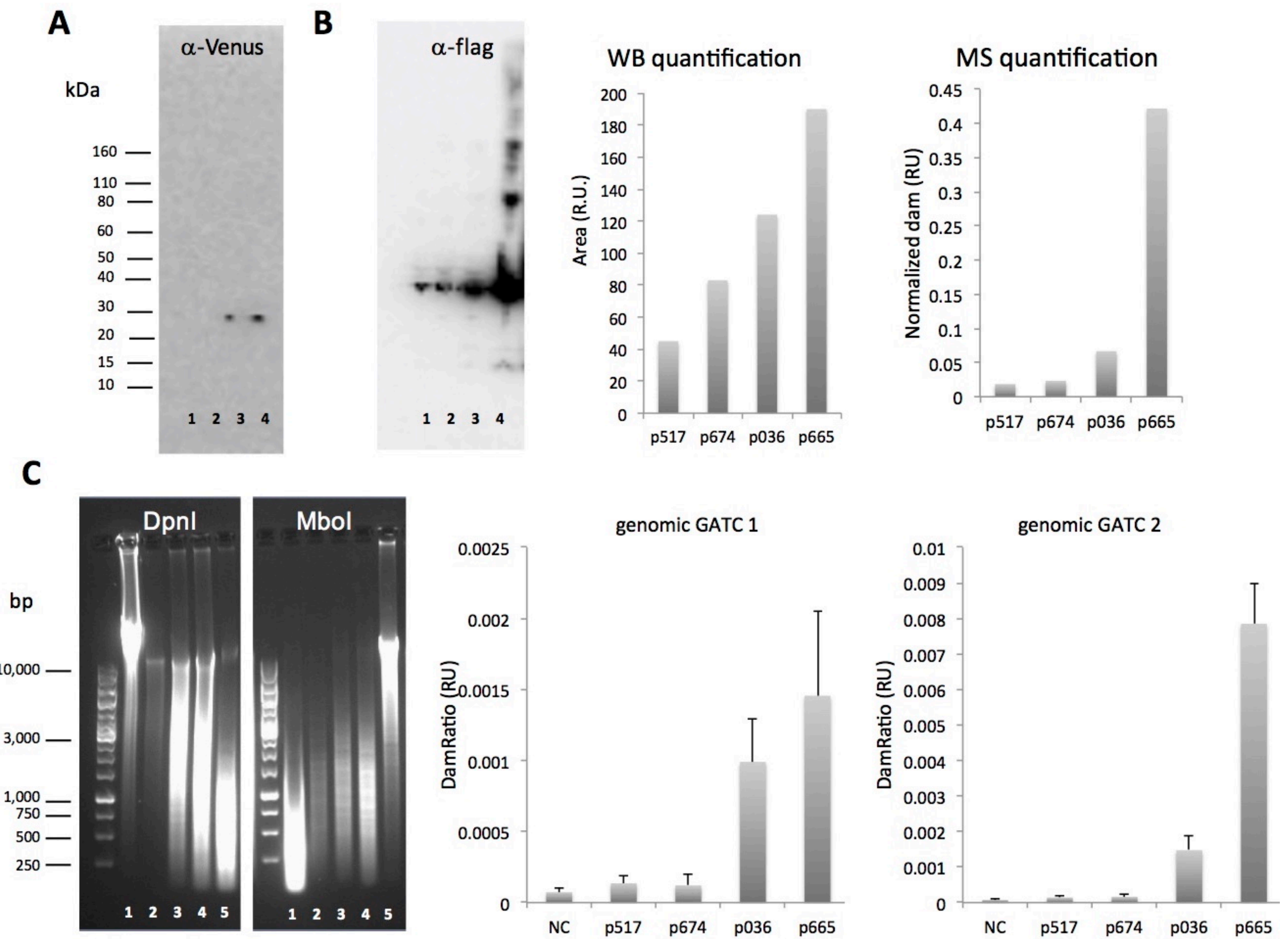
A
α-Venus
kDa
160
110
80
60
50
40
30
20
15
10
1 2 3 4
B
α-flag
1 2 3 4
WB quantification
Area (R.U.)
p517 p674 p036 p665
MS quantification
Normalized dam (RU)
p517 p674 p036 p665
C
DpnI
MboI
bp
10,000
3,000
1,000
750
500
250
1 2 3 4 5
1 2 3 4 5
genomic GATC 1
DamRatio (RU)
NC p517 p674 p036 p665
genomic GATC 2
DamRatio (RU)
NC p517 p674 p036 p665

Supplementary Figure 1. Proof of principle. A set of promoters of increasing strengths (from left to right in each panel) that are routinely used in the lab were chosen for the proof of principle (see also Methods). (**a**) The four endogenous promoters (1: MPN517, 2: MPN674, 3: MPN036 and 4: MPN665) were cloned in front of a YFP-Venus reporter, expressed in *M. pneumoniae* and proteins were extracted and probed with anti-GFP antibody. The molecular weight ladder was Novex Sharp Pre-stained Protein Standard (Thermo Fisher Scientific). (**b**) The same promoters were cloned to drive dam-Flag expression. In the left panel, the anti-Flag tag Western blot is shown. The middle panel shows the quantification of the Dam signal. The right panel is the quantification by LC-MSMS (normalized with MPN001, see Supplementary Data 2 and copy number in Supplementary Table 6). (**c**) Genomic DNA was extracted from cells shown in (**b**) and 1 µg was independently digested with either DpnI (cuts $G^{m}ATC$) or MboI (GATC), before running it on a 1% agarose gel (left panels, 1: Non-expressing Dam control; 2: MPN517, 3: MPN674, 4: MPN036 and 5: MPN665 promoters). The molecular weight ladder was 1kb Gene Ruler from MBI-Fermentas. GATC methylation was determined by doing qPCR of GATCs at two different locations in the genome (genomic GATC-1 is at coordinate 19,580 and GATC-2 at 170,908) of the two digestions shown in the gels (ratio of MboI/DpnI is plotted in the two right panels; AU: arbitrary units). As shown, Dam protein and methylation correlate well. This means that the DAMRatio reflects the protein abundance and is therefore a quantitative reporter. Error bars show the standard deviation (n=3).

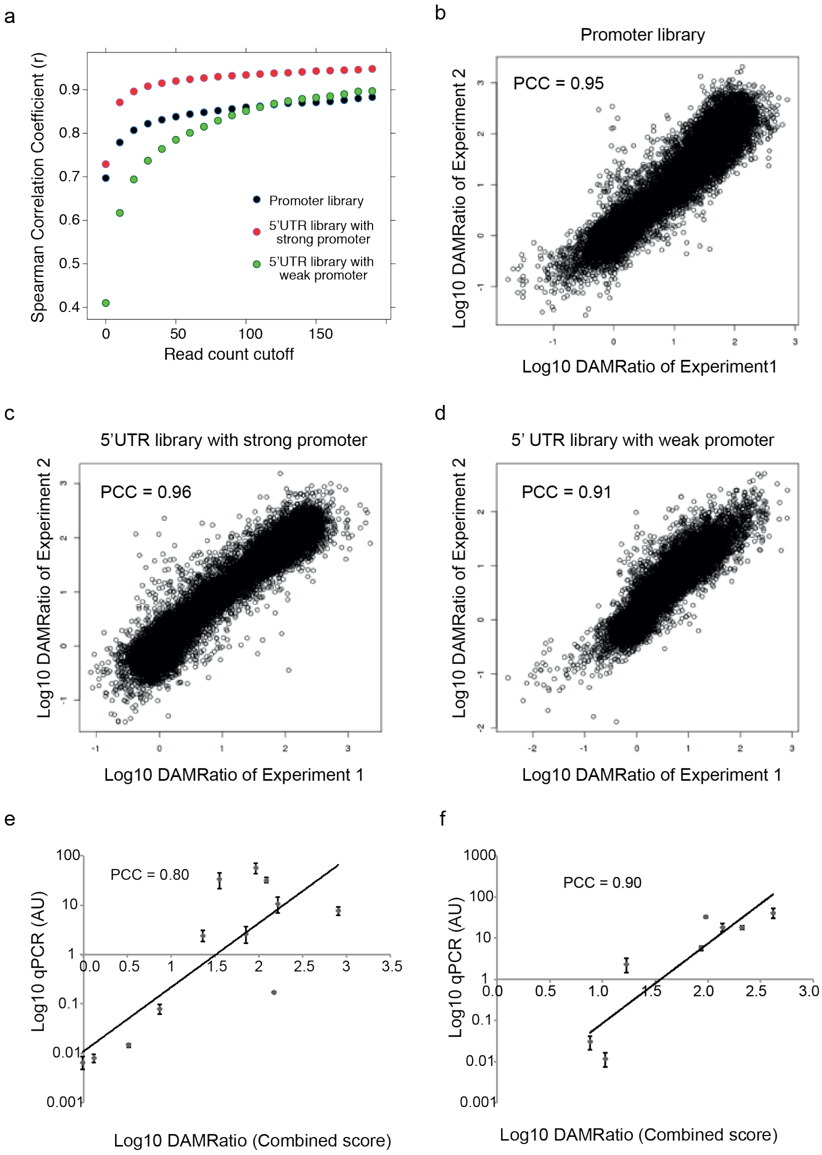

Supplementary Figure 2. Correlation between two screening replicas and validation of DAMRatios by quantitative PCR. (**a**) Correlation between two biological replicates according to read count cutoff (see Supplementary Table 4). (**b, c, d**) The correlations of the DAMRatios between two biological replicates when using a read count cutoff of 100. Transcription study and 5'-UTR study with strong and weak promoters are highly reproducible. The Pearson Correlation Coefficient (PCC) between replicates is reported. (**e, f**) DAMRatios reflect the Dam activity validated by methylation-sensitive qPCR. (**e**) From the transcription study, twelve selected promoters were individually cloned and validated (Supplementary Table 5). DNA activity was measured by qPCR (x-axis). (**f**) DAMRatio indicates the activity of Dam estimated from the Translation study; eight selected 5'-UTRs with the strong promoter were individually cloned (Supplementary Table 5). (**e, f**) The PCC between replicates is reported. qPCR values are in arbitrary units (AU). See sequences in Supplementary Table 2. Error bars show the standard deviation (n=3).

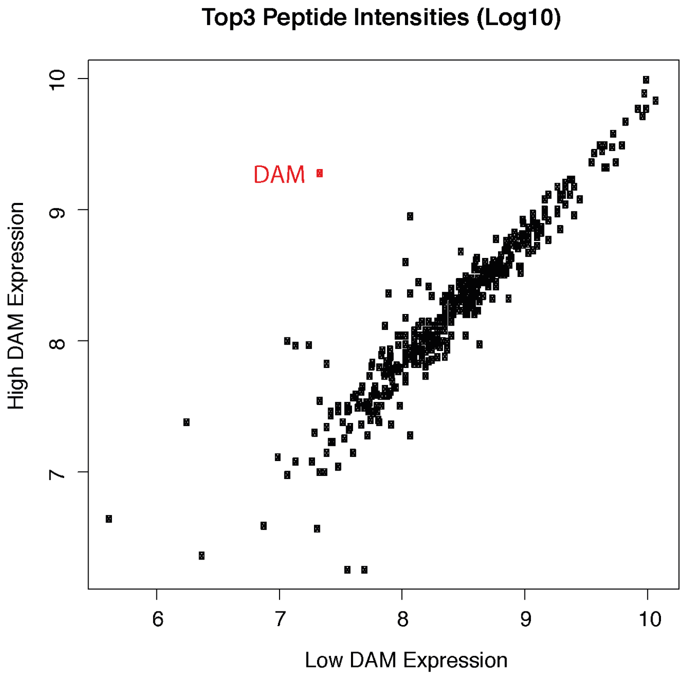

Supplementary Figure 3. Proteome comparison between low and high expression of Dam protein. We compare the log10 of the average Top 3 peptides intensities of protein extracts from low (promoter p4, estimated ~1 protein copy per cell) and high expression (Promoter p1, estimated ~2,300 protein copies per cell) of Dam protein constructs (see Supplementary Tables 2 and 5). The red spot is the Top 3 peptides intensities of Dam protein. When we take three constructs expressing high Dam with similar values (p6, UTR1 and 2) and three that have no detectable levels (p5, p12 and UTR8), we obtained only 34 proteins with a *P*-value lower than 0.05. This within the noise (with 494 proteins and a false discovery rate, FDR of 0.1, we could get 20 proteins).

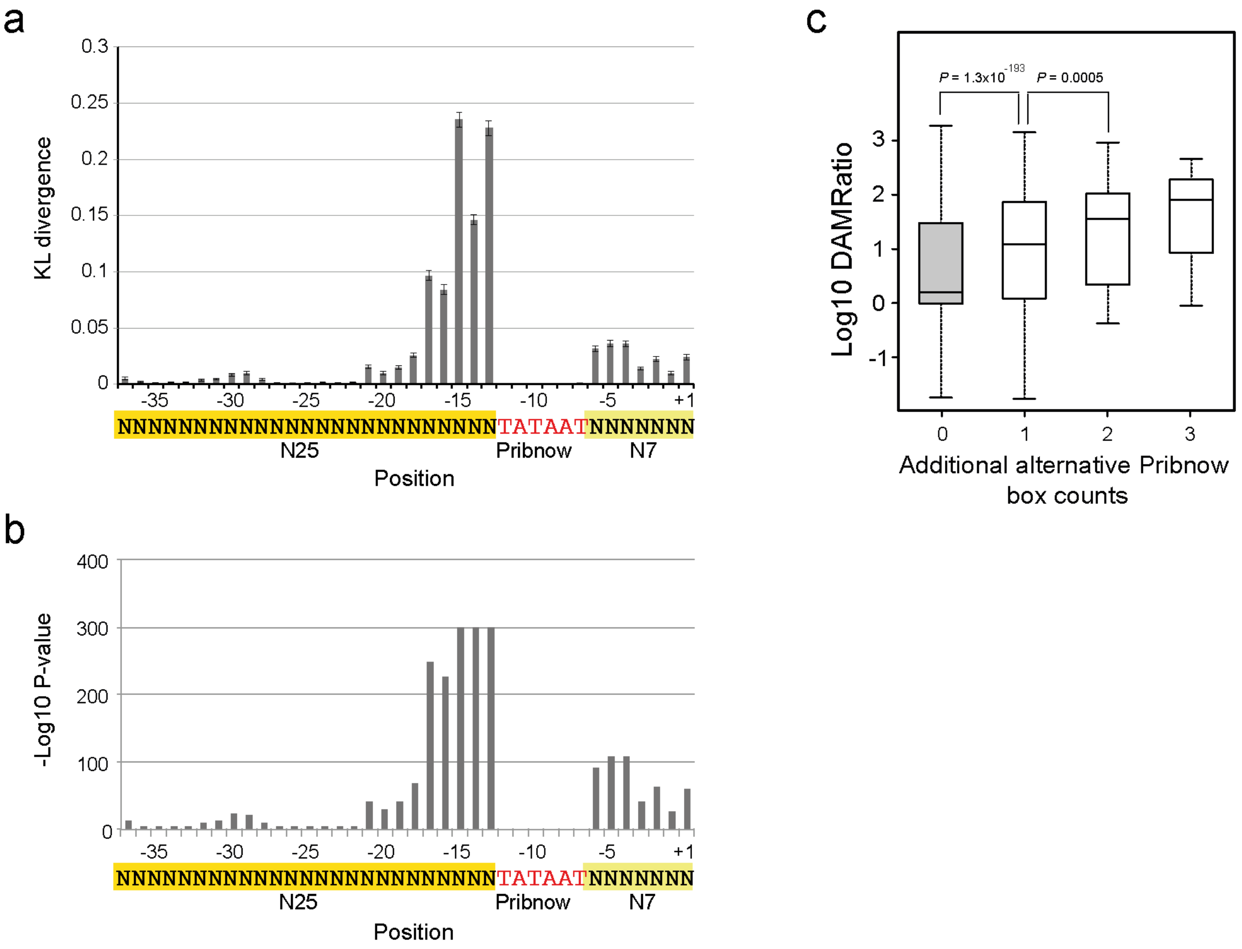

Supplementary Figure 4. Promoter region position-wide relative impacts on promoter strength and effects of tandem Pribnow motifs. (**a**) Kullback-Liebler (KL) divergence and (**b**) *P*-values indicate the difference of nucleotide distribution from background distributions in a specific position between productive promoters. The upstream proximal region of the Pribnow box shows remarkable differences when compared to other positions. The downstream proximal region of the Pribnow box as well as the -30 region also have a significant impact on promoter strength ($P = 3.9 \times 10^{-24}$ at -30). *P*-values were calculated using the *Chi*-squared test. (**c**) Effects of tandem alternative Pribnow box motifs. The DAMRatio found for additional alternative Pribnow motifs (TAAAAT, TACAAT, TAGAAT, and TATAAT) in the randomized promoters proportionally increases with the number of alternative Pribnow motifs.

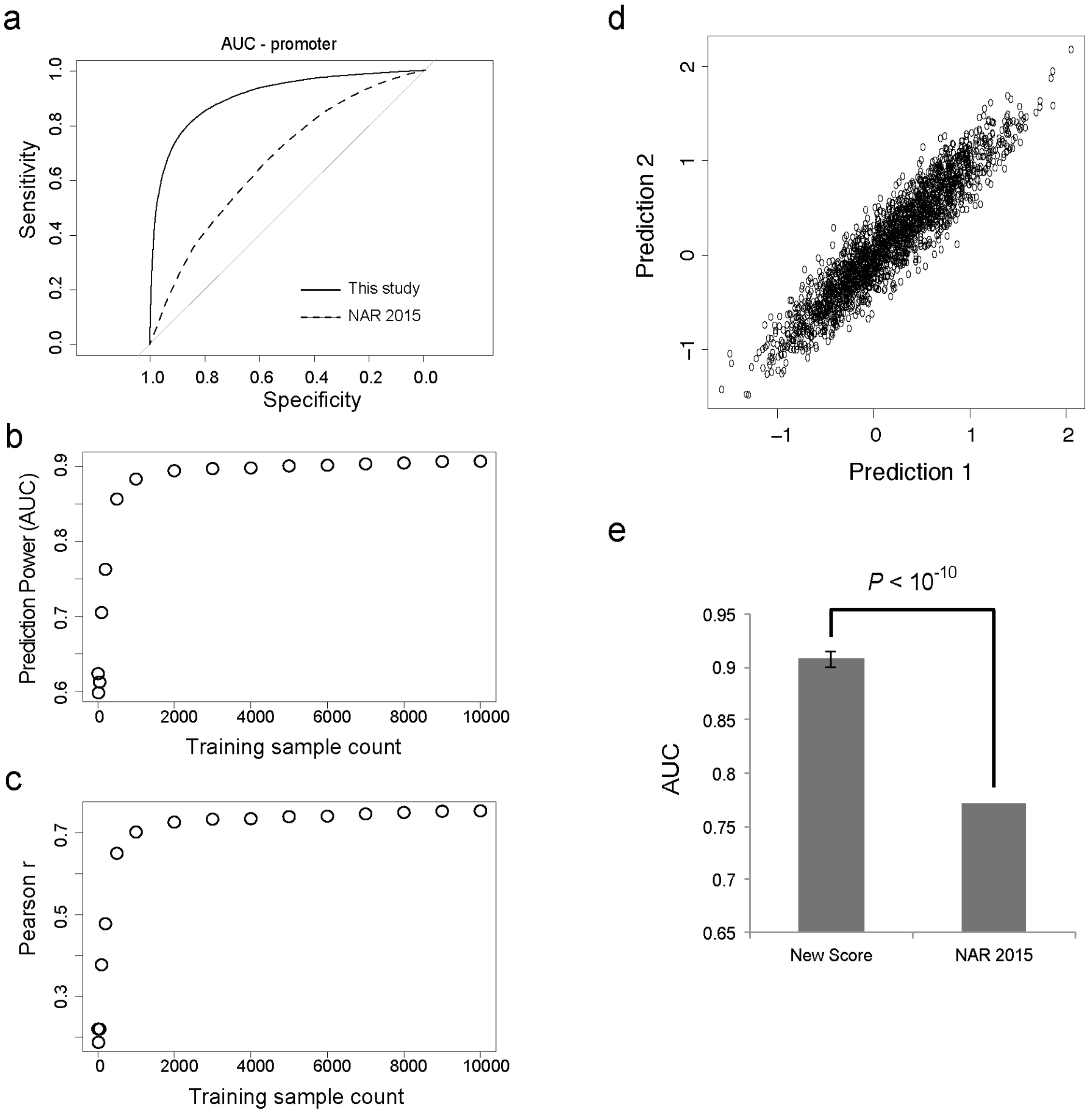

Supplementary Figure 5. Prediction of promoter strength and training sample saturation analysis. (a) Promoter prediction power of this study and of the previously published random forest scoring method[6]. **(b)** Training sample saturation analysis with prediction power (AUC). We used different number of training sets and the same test set for the analysis. The prediction power (AUC) becomes saturated when the number of training samples reaches 2,000. **(c)** Training sample saturation analysis with Pearson correlation (*r*). The Pearson correlation coefficient between predicted promoter strengths and observed DAMRatios also becomes saturated when the number of training samples reaches 2,000. (**d**) Comparison between two predictions (from support vector regression) of promoter strength using different training sets but the same test set. The correlation between the two predictions is $r = 0.98$. The number of sequences used to train and test were 5,000 and 2,000, respectively. (**e**) Comparison of the promoter prediction accuracy with our previous predictor (NAR 2015[6]) from promoter-like

sequences (the cases containing the TANAAT Pribnow motif). The promoters and non-promoters are the same test set as in the published predictor[6], but contain the TANAAT sequence. Error bars show the standard deviation.

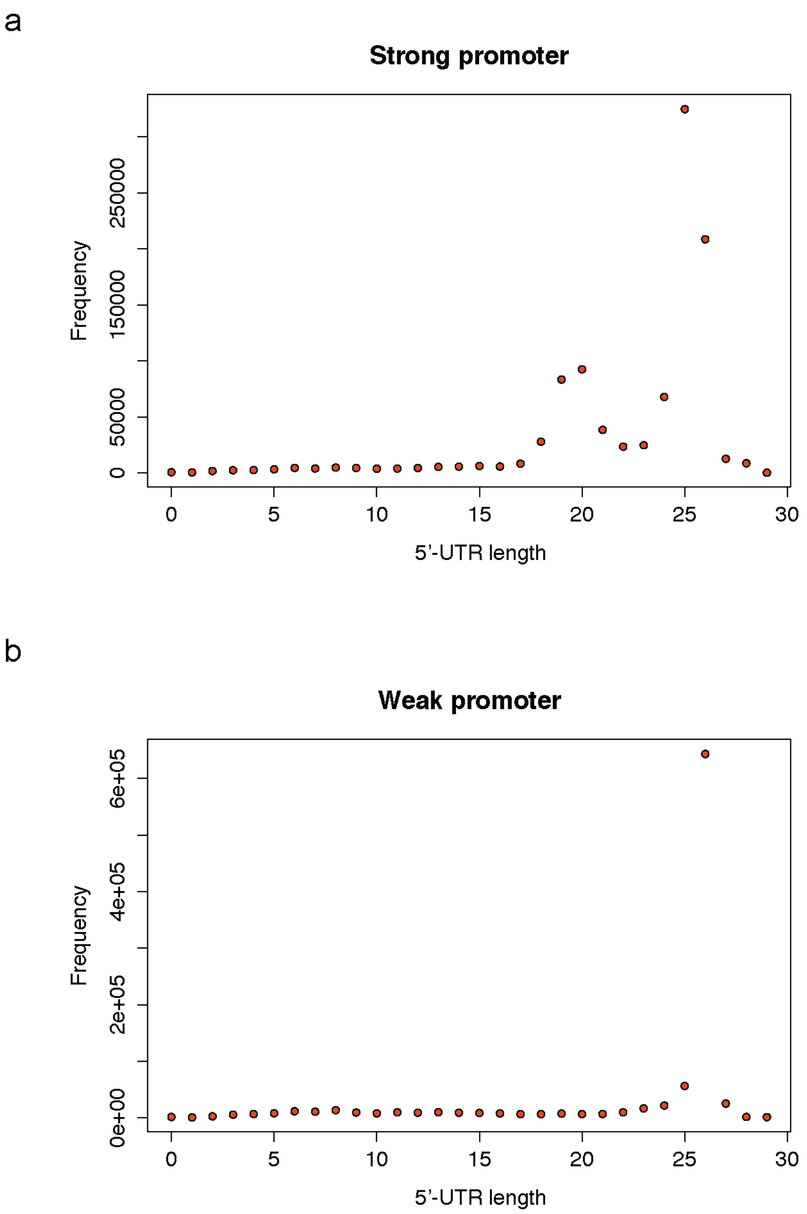

Supplementary Figure 6. 5'-UTR length distributions. (**a**) 5'-UTR lengths estimated from RNA-seq of the strong promoter screen. The majority of 5´-UTRs are 25 or 26 nt long. There is a small fraction of mRNAs however, that have 5'-UTRs of 20 nt, possibly arising from transcription of an alternative Pribnow box within the strong promoter. (**b**) 5'-UTR lengths estimated from RNA-seq of the weak promoter screen. In this case, transcripts with 26 nt 5´-UTRs were the predominant species found.

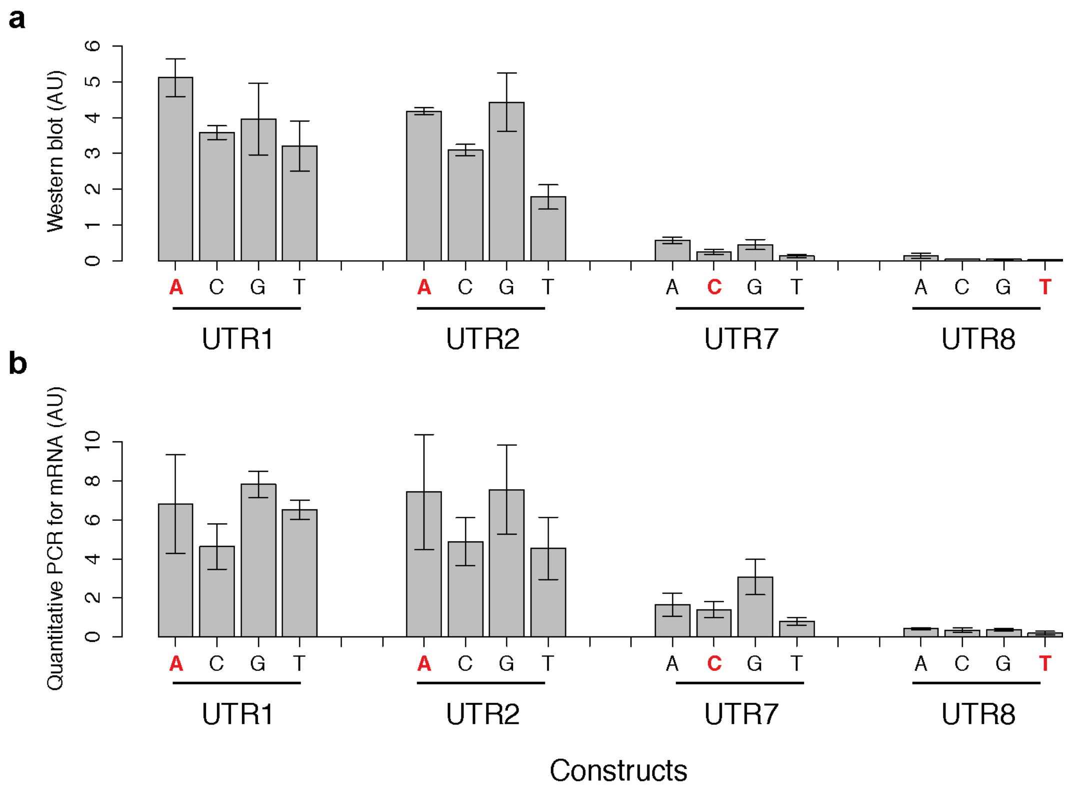

Supplementary Figure 7. Protein and mRNA abundance changes in function of the TSS. (**a**) Western blot intensities of all variant constructs (see Supplementary Tables 1, 2 and 12a). (**b**) mRNA abundance from quantitative PCR (see Supplementary Table 12a). AU means Arbitrary Units. (**a, b**) All mutants are driven by the strong promoter (Syp32, where N7 is a T; natural +1 position in the mRNA) and the mutated base is at N8. The original bases are indicated in bold red font. Note that the original UTR1 and UTR2 have high DAMRatios, while the original UTR7 and UTR8 have lower DAMRatios. In general, A and G bases are flavored for higher mRNA and protein levels. Error bars show standard deviation (n=3).

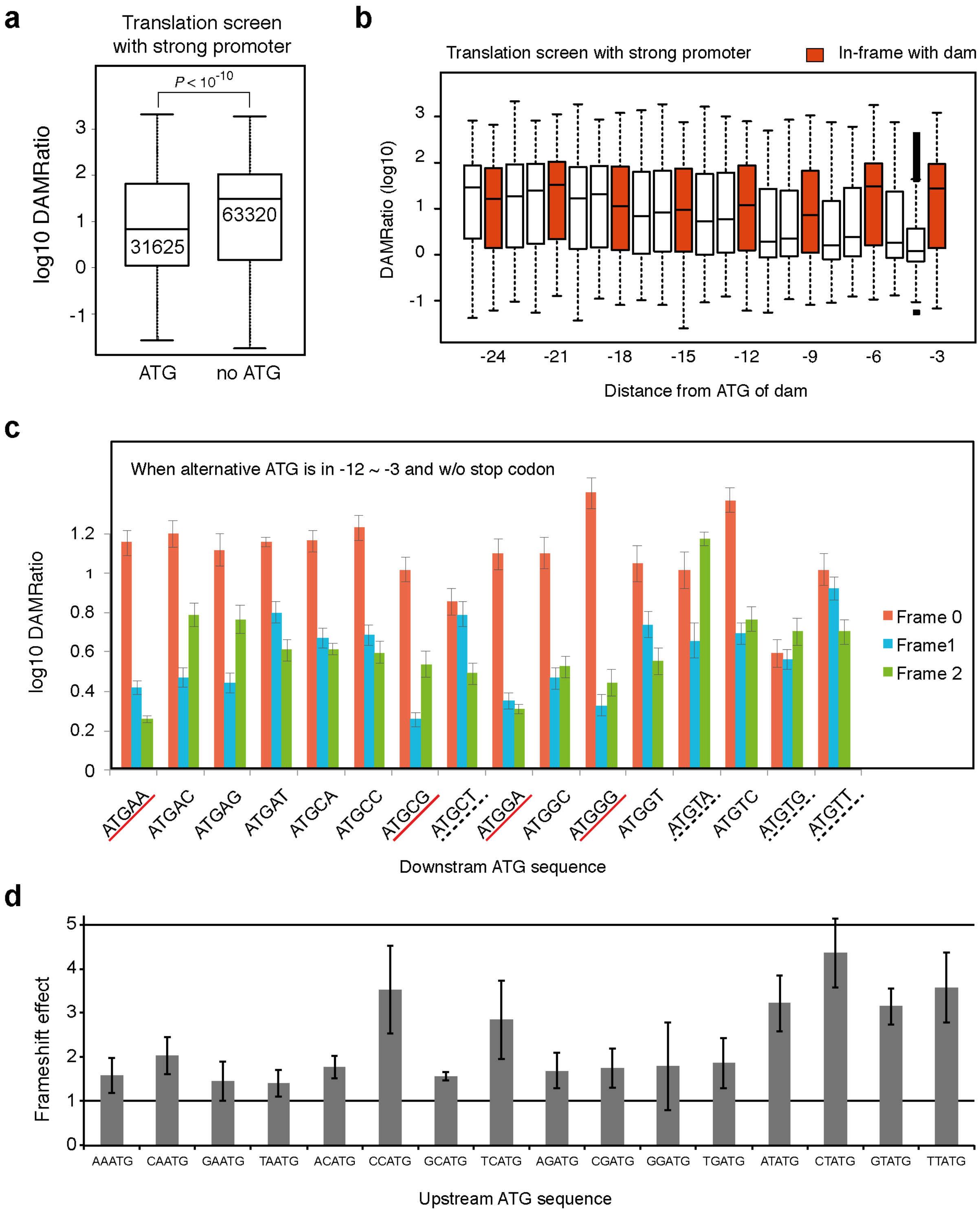

Supplementary Figure 8. The effect of alternative translation start sites. (**a**) Alternative translation start sites in the 5'-UTR systematically reduce the DAMRatio. "ATG" and "no ATG" depict the DAMRatio distributions of 5'-UTRs having alternative ATGs or not, respectively. (**b**) The frame-shift effect of alternative 5'-UTR translation start sites (ATG) on translation efficiency. When an ATG is located between positions -25 and -13, there is no significant reduction of translation efficiency by the alternative ATG. In contrast, when an ATG is located between

positions -12 and -3, the translation of the correct Dam frame is significantly reduced, but only when the ATG is located out-of-frame with respect to the actual ATG. **(c)** The effects of alternative translation start sites and their downstream sequences. The frame-shift effect is dependent on the down-stream nucleotides of the alternative ATG. For example, ATGAA and ATGGG have significantly different translation rates according to their frame, whereas ATGTT and ATGTG have similar translation rates regardless of their frame. **(d)** The effects of alternative translation start sites and their upstream sequences. The frame-shift effect is defined by: DAMRatio of in-frame / DAMRatio of out-of-frame. (**c, d**) All analyses were done removing any sequences bearing stop codons (TAA and TAG). Error bars show standard error.

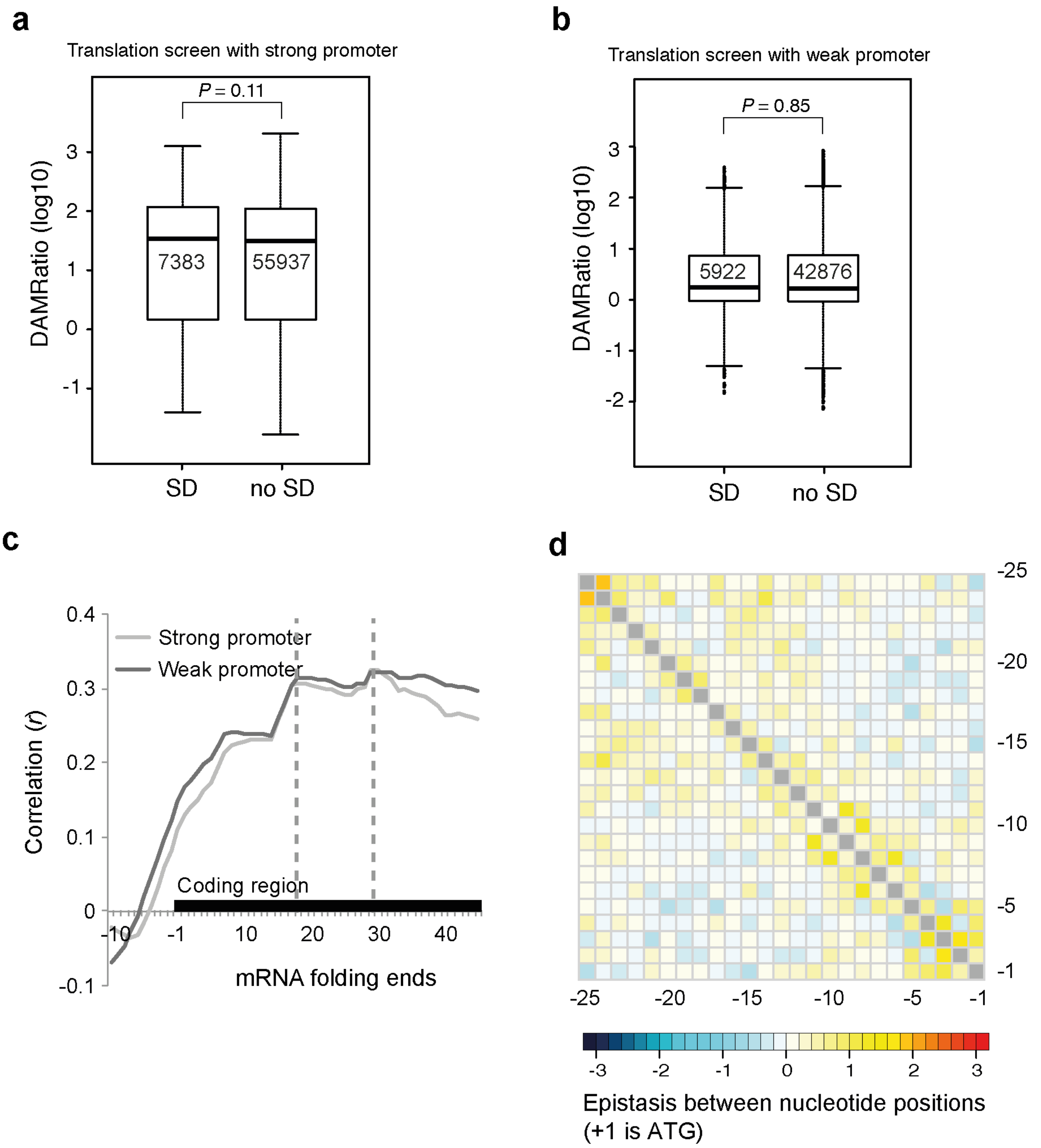

Supplementary Figure 9. The effect of various 5'-UTR features on translation. (**a, b**) DAMRatios according to the presence of Shine-Dalgarno (SD)-like sequences (GAGG, GGAG, AGGA and GAAG) in the 5'-UTRs. Only SDs located up to base -20 before the ATG were considered. SD sequences were scanned in the strong (**a**) and weak promoter screens (**b**). Sequences having alternative ATGs in their 5'-UTR were excluded from the analysis. (**c**) Influence of universal RNA structural features on translation efficiency. With the mRNA folding start position fixed at -25, the end position of folding was varied from position -10 to +45 in order to identify folding regions (see Supplementary Data 5). An increase in the correlation between folding energy and Dam level (0.3 for weak and 0.32 for strong promoter) was observed until it reached a plateau after nucleotide +18. (**d**) Epistatic interactions between nucleotide positions of the 5'-UTRs from the weak promoter screen (see Methods).

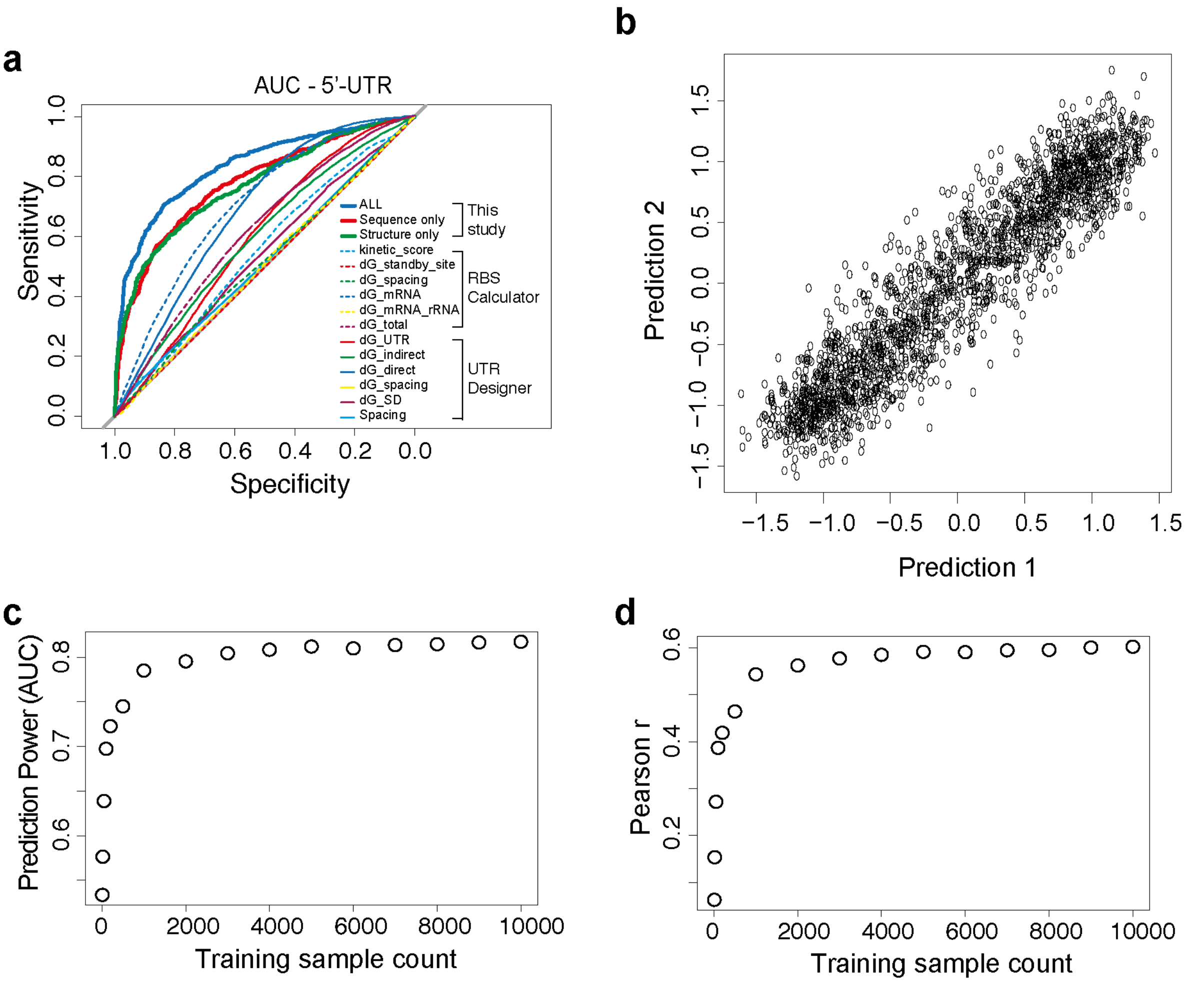

Supplementary Figure 10. Prediction of 5'-UTR translation efficiency and training sample saturation analysis. (a) 5'-UTR translation efficiency prediction power of this study and the two well-known predictors, RBS Calculator and UTR Designer. (**b**) Comparison of two translation efficiency predictions using different training sets with the same test set. The correlation between the two predictions was r = 0.93. The number of sequences used to train and test were 5,000 and 2,000, respectively. All the sequences in the test and training sets are different. **(c**) Training sample saturation analysis with prediction power (AUC). We used a different number of training sets and the same test set for the analysis. **(d**) Training sample saturation analysis with Pearson correlation (r). (**c, d**) The prediction power (AUC) and Pearson correlation coefficient between predicted promoter strengths and observed DAMRatios become saturated when the number of training samples reaches 2,000.

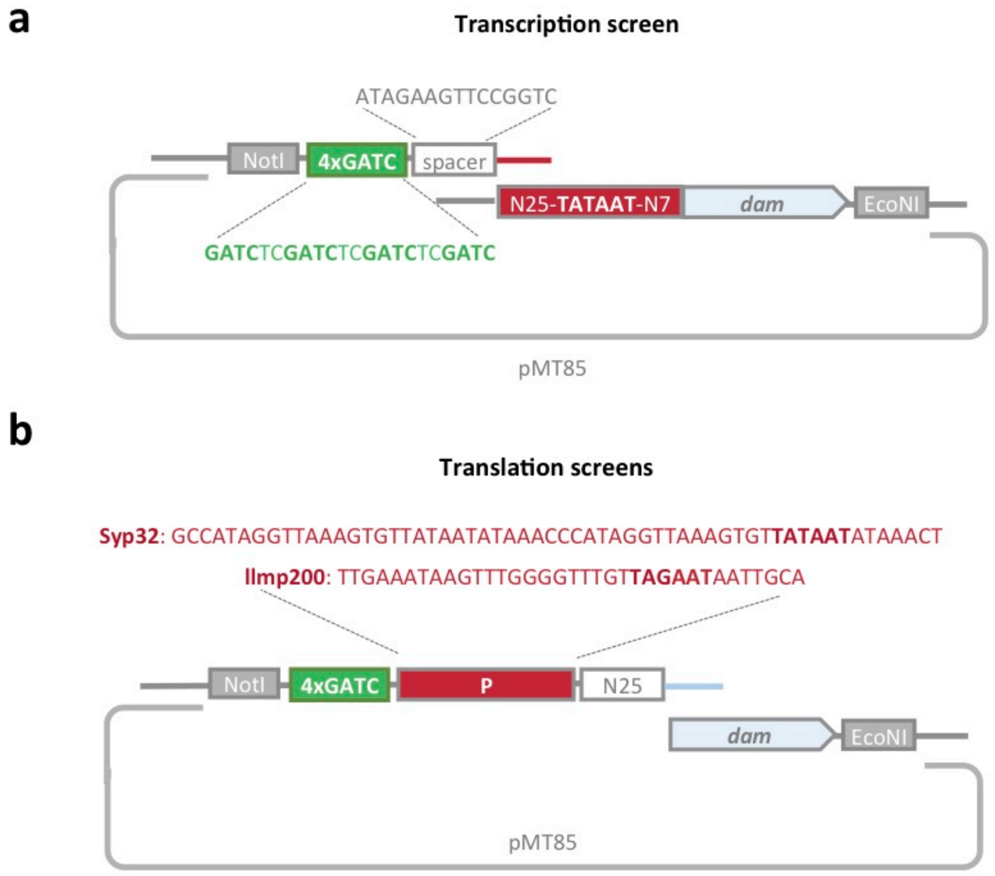

Supplementary Figure 11. Gibson assembly cloning of the screening constructs. A 3-fragment Gibson Assembly (GA) mix was prepared with a NotI-EcoNI cut vector, a linker and a PCR product. (**a**) In the transcription screen, a spacer was included in order to avoid methylation interfering with RNA polymerase binding to the promoter. (**b**) In the translation screen, there are actually 2 GAs, one with a strong (SyP32) and the other with a weak (Il_mp200) promoter (see Methods). The overlapping regions are shown one over the other.

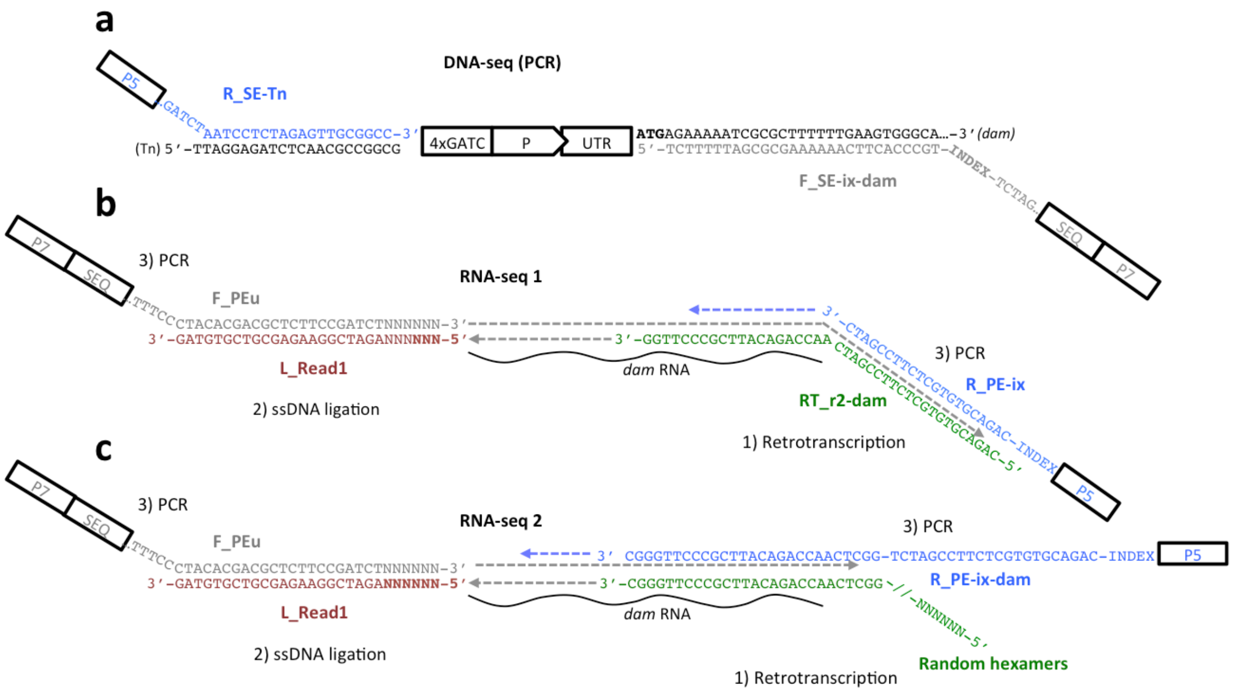

Supplementary Figure 12. Library sequencing scheme. (**a**) DNA-seq was performed by PCR amplification of the screen cassette. The custom PCR oligos included the Illumina sequencing, flow-cell binding and index sequences. (**b**) RNA-seq was possible only for the 5'-UTR screen. The first protocol is a variation of SHAPE-seq that includes a specific amplification of the 5' *dam* mRNA. There was an additional enrichment step after the RT (see Methods). (**c**) The second RNA-seq approach uses standard RT with random hexamers, and a dam-specific PCR step (see Methods).

a

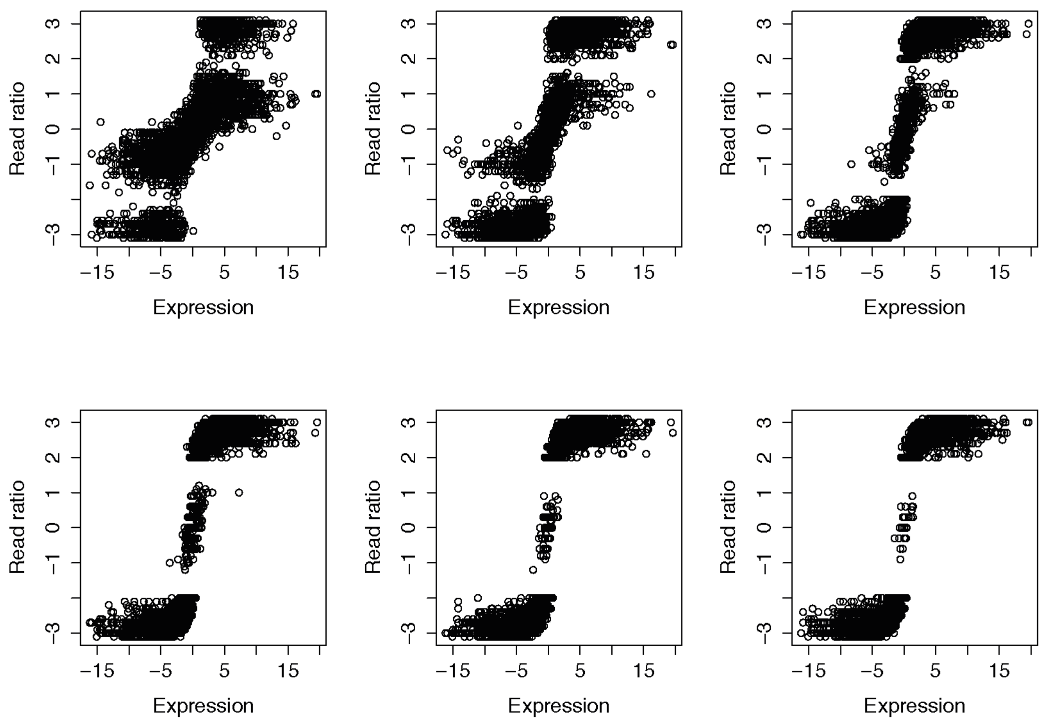

b

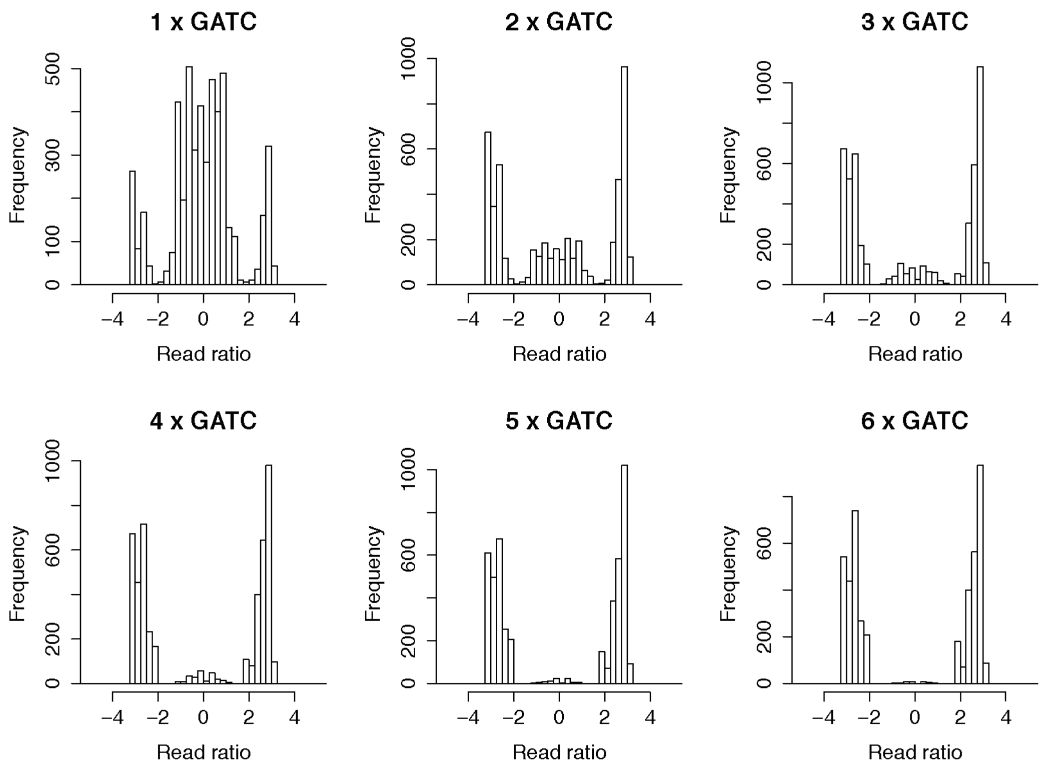

Supplementary Figure 13. Simulation results of the read ratio distribution of Dam activity. (a) Correlation of gene expression and simulated DAMRatio. (b) Distribution of simulated DAMRatios according to number of GATC sites. The input distribution of gene expression was assumed to follow a normal distribution. Trimodal distributions with various proportions of the

second peak were obtained depending on the number of GATC sites. The efficiencies of both methylation-sensitive enzymes were set as 95% and 10 PCR cycles were considered. The initial number of isogenic constructs in the population was set as 10 with 1 standard deviation (Frequency in Arbitrary Units).

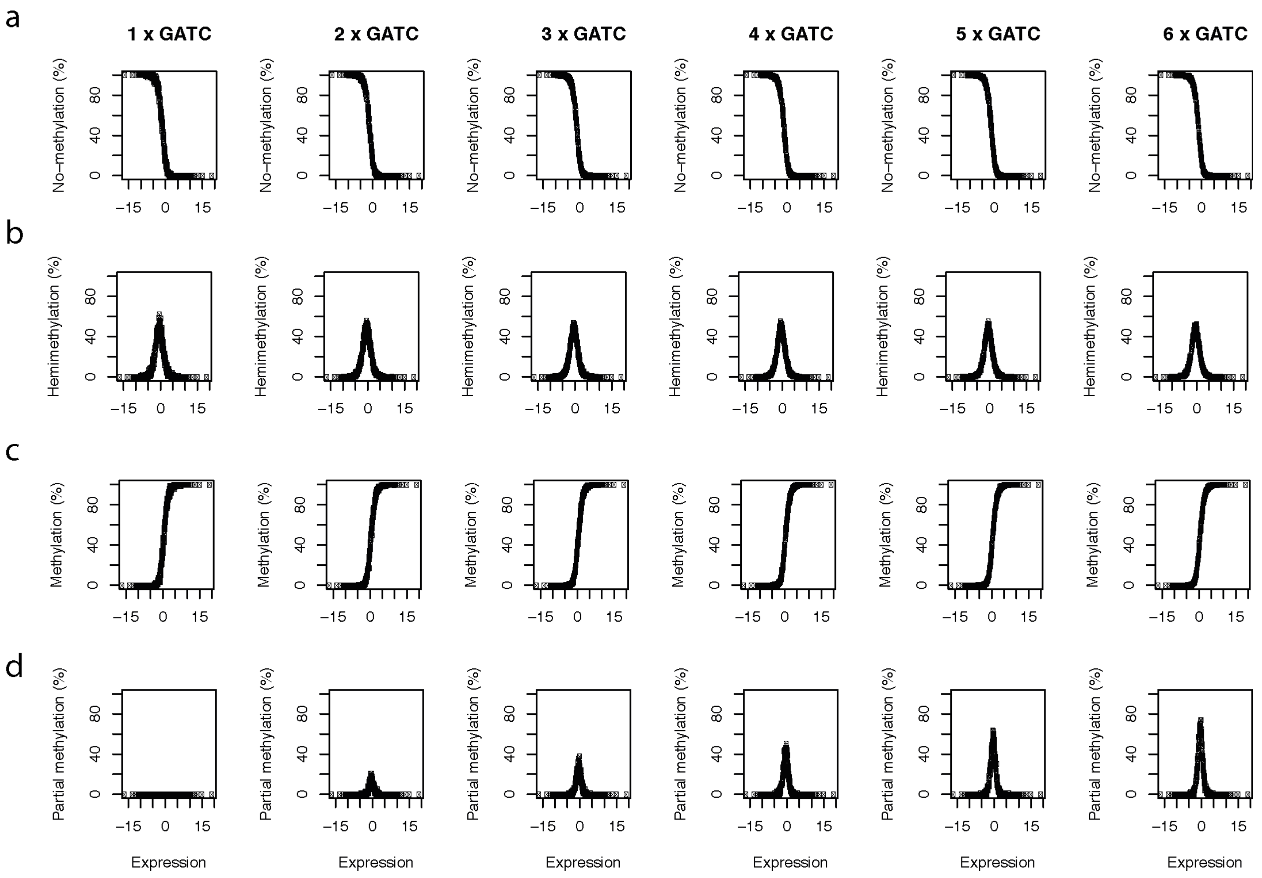

Supplementary Figure 14. Simulation results of the methylation status of GATC sites according to Dam expression. (**a**) Probability of no methylation per site. (**b**) Probability of hemimethylation per site. (**c**) Probability of no methylation per site. (**d**) Probability of partial methylation of GATC sites. The values of Dam expression are shown in the X axis in arbitrary units. The larger the number of GATC sites, the higher chance to have partial methylation under intermediate Dam expression values (near 0). To simulate the hemimethylated DNA, the methylation probability depending on Dam expression (arbitrary unit) was considered in each strand separately. To show statistically stable results for the figure, the initial number of isogenic constructs in the population was set as 100 with 1 standard deviation (frequency in Arbitrary Units).

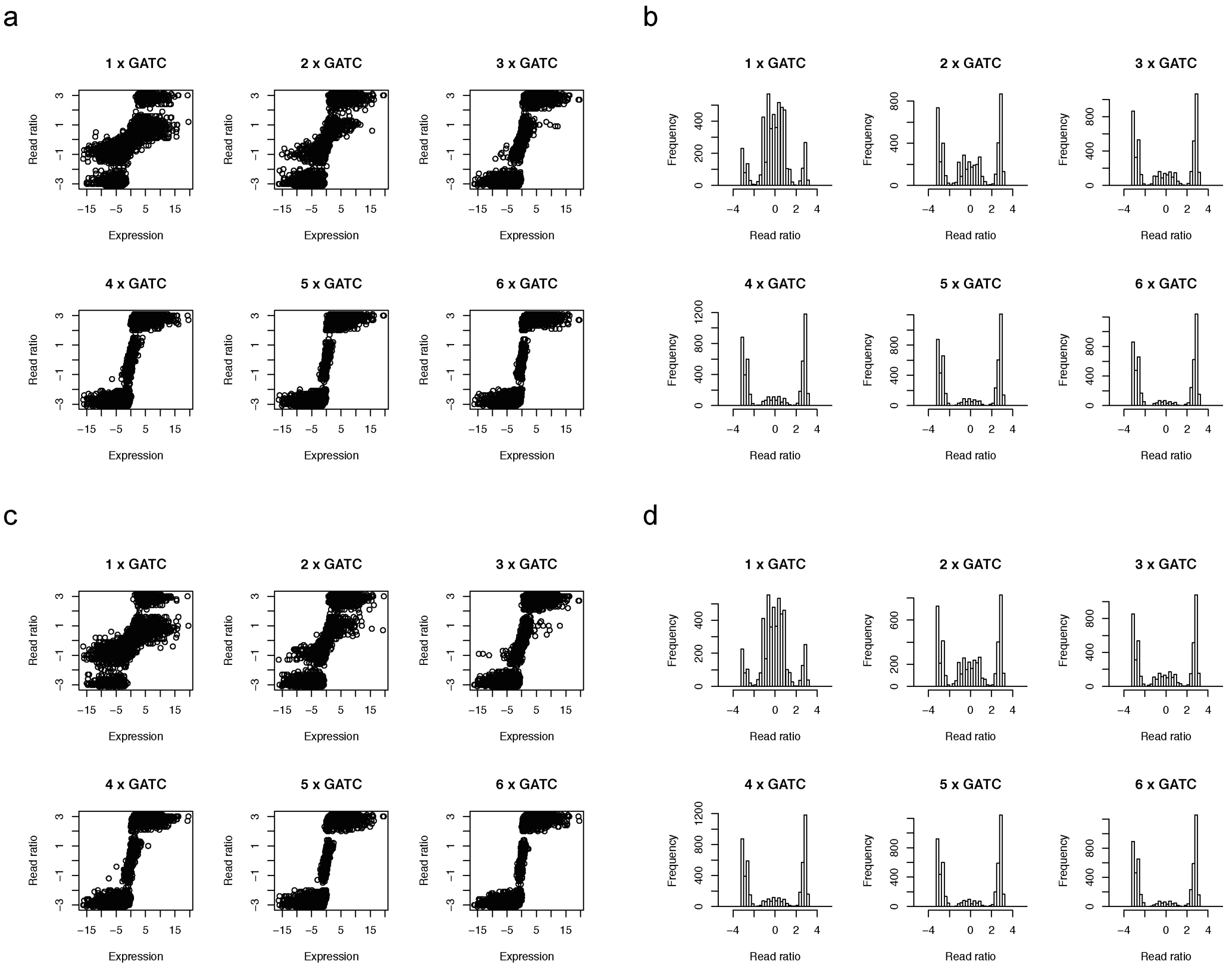

Supplementary Figure 15. Simulation results of the read ratio distribution of Dam activity considering hemimethylation. (**a**, **c**) Correlation of gene expression and simulated DAMRatio. (**b**, **d**) Distribution of simulated DAMRatios according to number of GATC sites. (**a**, **b**) When DpnI and MboI can cut 2% of hemimethylated DNA. (**c**, **d**) When DpnI can cut (2% activity) but MboI cannot cut hemimethylated DNA. All the simulation procedures were the same as in Supplementary Fig. 13 except for including hemimethylated DNA status and corresponding enzyme activity. To consider hemimethylated DNA the methylation probability depending on Dam expression (arbitrary unit) was considered independently for each strand.

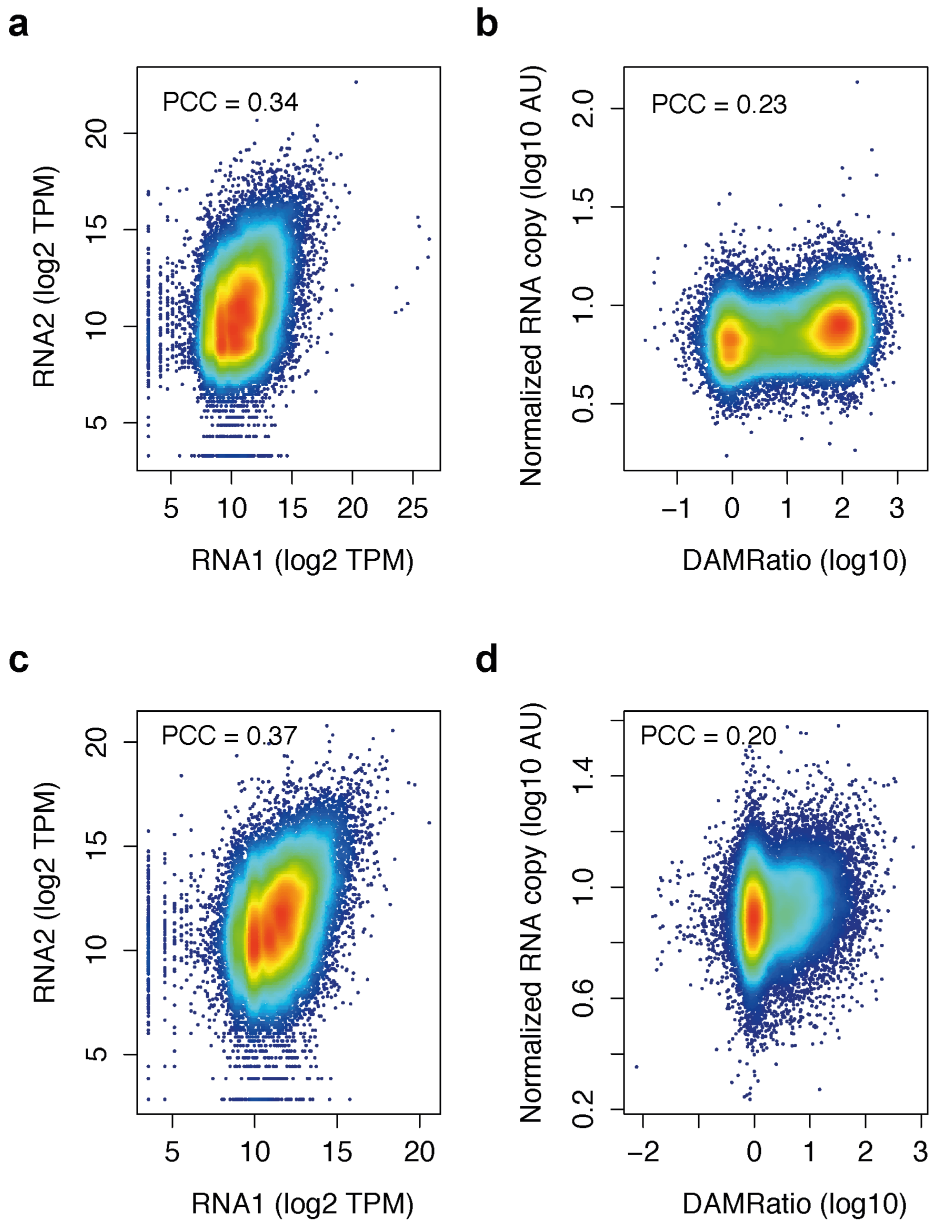

Supplementary Figure 16. Comparison of DAMRatios and mRNA copy numbers in 5'-UTR libraries having strong promoter and weak promoters. (**a, b**) 5'-UTR library with strong promoter setup. (**c, d**) 5'-UTR library with weak promoter setup. (**a, c**) mRNA copy numbers were obtained from two Dam-specific RNA-seq experiments ("RNA1" and "RNA2"). Two different RNA-seq experiments significantly correlated (both cases $P < 2.2 \times 10^{-16}$). (**b, d**) The RNA copy numbers were normalized by DNA copy numbers. Correlation of DAMRatio and mRNA copy number is also significant (both cases $P < 2.2\text{x}10^{-16}$). (**a, b, c, d**) The "heat" colored blue to red corresponding to the plotting density. The Pearson Correlation Coefficient (PCC) between replicates is reported.

Supplementary Table 1. Plasmids used in this study

Vectors, fragments and cloning strategy are indicated. For PCR, genomic DNA was used as template. Clones follow an internal plasmid codification system. V means vector and F means fragment. # pMT85-tuf->Venus (R70-3) was published in Yus *et al* ., 2012. pGEM-T Easy was purchased from Promega. Primers are listed in Supplementary Data 1.

Project	Short	Vector	V Restriction	F 1	F Restriction	Forward oligo	Reverse oligo	F 2	Forward oligo	Reverse oligo	Strategy	Clone
pMT85-tuf->MCS	p665-Venus	R70-3#	KpnI+EcoNI	Linker	KpnI+EcoNI	F_MCS_pMT	R_MCS_pMT				Linker	E240-2
pMT85-p674-Venus	p674-Venus	R70-3#	NotI+Acc65I	Linker		F_ldhp1_Not	R_ldhp1_Acc65				Linker	E369-22
pGEM-T-Easy-NotI-p036-Acc65I		pGEM-T				F_p036_Not	R_p036_Acc65				AT cloning	E386-5
pMT85-p036-Venus	p036-Venus	R70-3#	NotI+Acc65I	E386-1	NotI+Acc65I						Restriction	E384-7
pMT85-p517->Venus	p517-Venus	R70-3#	NotI+Acc65I	Linker	NotI+Acc65I	F_p517_Not	R_p517_Acc				Linker	E458-7
pMT85-tuf->flag-MCS		E240-2	NsiI+EcoRV	Linker		F_flag_Nsi	R_flag_RV				Linker	E630-17
pGEM-dam		pGEM-T				F_dam_Acc65	R_dam_Nsi				AT cloning	E897-1
pGEM-dam*		E897-1				F_dam_Nsi*	R_dam_Nsi*				Mutagenesis	E907-19
pMT85-tuf->dam-flag	p665-dam	E630-17	Acc65+NsiII	E907-19	Acc65+NsiII						Restriction	E908-1
pMT85-p674->dam-flag	p674-dam	E908-1	NotI+Acc65	Linker		F_ldhp1_Not	R_ldhp1_Acc65				Linker	E909-1
pMT85-p036->dam-flag	p036-dam	E908-1	NotI+Acc65	E386-5	NotI+Acc65						Restriction	E910-4
pMT85-p517->dam-flag	p5175-dam	E908-1	NotI+Acc65	Linker		F_p517_Not	R_p517_Acc				Linker	E911-6
pMT85-4GATC-Prom->Dam		R70-3#	NotI + EcoNI	Linker		F_pMT_4GATC	R_pMT_4GATC	PCR	F_prom_Dam	R_Dam_pMT	Gibson	Libraries
pMT85-4GATC-Syp32->UTR25-Dam		R70-3#	NotI + EcoNI	Linker		F_pMT_Syp32	R_Syp32_Dam	PCR	F_Dam	R_Dam_pMT	Gibson	Libraries
pMT85-4GATC-llmp200->UTR25-Dam		R70-3#	NotI + EcoNI	Linker		F_pMT_llmp200	R_llmp200_Dam	PCR	F_Dam	R_Dam_pMT	Gibson	Libraries
pMT85-Syp32->dam-flag	SyP32-dam	E908-1	NotI+Acc65	Linker		F_SyP32	R_Syp32_Dam				Linker	E1031-7
pMT85-llmp200->dam-flag	llmp200-dam	E908-1	NotI+Acc65	Linker		F_LLmp200	R_LLmp200				Linker	E1032-10
pMT85-4GATC-p1->dam	p1-dam	R70-3#	NotI + EcoNI	Linker		F_pMT_4GATC	R_pMT_4GATC	PCR	F_p1_dam	R_Dam_pMT	Gibson	E1107-7
pMT85-4GATC-p2->dam	p2-dam	R70-3#	NotI + EcoNI	Linker		F_pMT_4GATC	R_pMT_4GATC	PCR	F_p2_dam	R_Dam_pMT	Gibson	E1108-10
pMT85-4GATC-p3->dam	p3-dam	R70-3#	NotI + EcoNI	Linker		F_pMT_4GATC	R_pMT_4GATC	PCR	F_p3_dam	R_Dam_pMT	Gibson	E1109-13
pMT85-4GATC-p4->dam	p4-dam	R70-3#	NotI + EcoNI	Linker		F_pMT_4GATC	R_pMT_4GATC	PCR	F_p4_dam	R_Dam_pMT	Gibson	E1110-16
pMT85-4GATC-p5->dam	p5-dam	R70-3#	NotI + EcoNI	Linker		F_pMT_4GATC	R_pMT_4GATC	PCR	F_p5_dam	R_Dam_pMT	Gibson	E1111-19
pMT85-4GATC-p6->dam	p6-dam	R70-3#	NotI + EcoNI	Linker		F_pMT_4GATC	R_pMT_4GATC	PCR	F_p6_dam	R_Dam_pMT	Gibson	E1112-23

pMT85-4GATC-p7->dam	p7-dam	R70-3#	NotI + EcoNI	Linker		F_pMT_4GATC	R_pMT_4GATC	PCR	F_p7_dam	R_Dam_pMT	Gibson	E1113-10
pMT85-4GATC-p8->dam	p8-dam	R70-3#	NotI + EcoNI	Linker		F_pMT_4GATC	R_pMT_4GATC	PCR	F_p8_dam	R_Dam_pMT	Gibson	E1114-13
pMT85-4GATC-p9->dam	p9-dam	R70-3#	NotI + EcoNI	Linker		F_pMT_4GATC	R_pMT_4GATC	PCR	F_p9_dam	R_Dam_pMT	Gibson	E1115'-23
pMT85-4GATC-p10->dam	p10-dam	R70-3#	NotI + EcoNI	Linker		F_pMT_4GATC	R_pMT_4GATC	PCR	F_p10_dam	R_Dam_pMT	Gibson	E1116-20
pMT85-4GATC-p11->dam	p11-dam	R70-3#	NotI + EcoNI	Linker		F_pMT_4GATC	R_pMT_4GATC	PCR	F_p11_dam	R_Dam_pMT	Gibson	E1117-22
pMT85-4GATC-p12->dam	p12-dam	R70-3#	NotI + EcoNI	Linker		F_pMT_4GATC	R_pMT_4GATC	PCR	F_p12_dam	R_Dam_pMT	Gibson	E1118-25
pMT85-4GATC-Syp32->u1-dam	UTR1-dam	R70-3#	NotI + EcoNI	Linker		F_pMT_Syp32	R_u1_dam	PCR	F_dam	R_Dam_pMT	Gibson	E1151-7
pMT85-4GATC-Syp32->u2-dam	UTR2-dam	R70-3#	NotI + EcoNI	Linker		F_pMT_Syp32	R_u2_dam	PCR	F_dam	R_Dam_pMT	Gibson	E1152-10
pMT85-4GATC-Syp32->u3-dam	UTR3-dam	R70-3#	NotI + EcoNI	Linker		F_pMT_Syp32	R_u3_dam	PCR	F_dam	R_Dam_pMT	Gibson	E1153-15
pMT85-4GATC-Syp32->u4-dam	UTR4-dam	R70-3#	NotI + EcoNI	Linker		F_pMT_Syp32	R_u4_dam	PCR	F_dam	R_Dam_pMT	Gibson	E1154-16
pMT85-4GATC-Syp32->u5-dam	UTR5-dam	R70-3#	NotI + EcoNI	Linker		F_pMT_Syp32	R_u5_dam	PCR	F_dam	R_Dam_pMT	Gibson	E1155-19
pMT85-4GATC-Syp32->u6-dam	UTR6-dam	R70-3#	NotI + EcoNI	Linker		F_pMT_Syp32	R_u6_dam	PCR	F_dam	R_Dam_pMT	Gibson	E1156-22
pMT85-4GATC-Syp32->u7-dam	UTR7-dam	R70-3#	NotI + EcoNI	Linker		F_pMT_Syp32	R_u7_dam	PCR	F_dam	R_Dam_pMT	Gibson	E1157-22
pMT85-4GATC-Syp32->u8-dam	UTR8-dam	R70-3#	NotI + EcoNI	Linker		F_pMT_Syp32	R_u8_dam	PCR	F_dam	R_Dam_pMT	Gibson	E1158-23
pMT85-SyP32->u1A-dam-flag	SyP32-UTR1-A-dam	R70-3#		Linker		F_pMT_Syp32	R_U1_A_dam	PCR	F_dam	R_dam_Nsi_Flag	Gibson	A17-1
pMT85-SyP32->u1C-dam-flag	SyP32-UTR1-C-dam	R70-3#		Linker		F_pMT_Syp32	R_U1_C_dam	PCR	F_dam	R_dam_Nsi_Flag	Gibson	A15-3
pMT85-SyP32->u1G-dam-flag	SyP32-UTR1-G-dam	R70-3#		Linker		F_pMT_Syp32	R_U1_G_dam	PCR	F_dam	R_dam_Nsi_Flag	Gibson	A14-6
pMT85-SyP32->u1T-dam-flag	SyP32-UTR1-T-dam	R70-3#		Linker		F_pMT_Syp32	R_U1_T_dam	PCR	F_dam	R_dam_Nsi_Flag	Gibson	A16-1
pMT85-SyP32->u2A-dam-flag	SyP32-UTR2-A-dam	R70-3#		Linker		F_pMT_Syp32	R_U2_A	PCR	F_dam	R_dam_Nsi_Flag	Gibson	A26-3
pMT85-SyP32->u2C-dam-flag	SyP32-UTR2-C-dam	R70-3#		Linker		F_pMT_Syp32	R_U2_C	PCR	F_dam	R_dam_Nsi_Flag	Gibson	A27-1
pMT85-SyP32->u2G-dam-flag	SyP32-UTR2-G-dam	R70-3#		Linker		F_pMT_Syp32	R_U2_G	PCR	F_dam	R_dam_Nsi_Flag	Gibson	A28-1
pMT85-SyP32->u2T-dam-flag	SyP32-UTR2-T-dam	R70-3#		Linker		F_pMT_Syp32	R_U2_T	PCR	F_dam	R_dam_Nsi_Flag	Gibson	A29-1
pMT85-SyP32->u7A-dam-flag	SyP32-UTR7-A-dam	R70-3#		Linker		F_pMT_Syp32	R_U7_A	PCR	F_dam	R_dam_Nsi_Flag	Gibson	A22-1
pMT85-SyP32->u7C-dam-flag	SyP32-UTR7-C-dam	R70-3#		Linker		F_pMT_Syp32	R_U7_C	PCR	F_dam	R_dam_Nsi_Flag	Gibson	A23-1
pMT85-SyP32->u7G-dam-flag	SyP32-UTR7-G-dam	R70-3#		Linker		F_pMT_Syp32	R_U7_G	PCR	F_dam	R_dam_Nsi_Flag	Gibson	A24-1
pMT85-SyP32->u7T-dam-flag	SyP32-UTR7-T-dam	R70-3#		Linker		F_pMT_Syp32	R_U7_T	PCR	F_dam	R_dam_Nsi_Flag	Gibson	A25-3
pMT85-SyP32->u8A-dam-flag	SyP32-UTR8-A-dam	R70-3#		Linker		F_pMT_Syp32	R_U8_A_dam	PCR	F_dam	R_dam_Nsi_Flag	Gibson	A18-3
pMT85-SyP32->u8C-dam-flag	SyP32-UTR8-C-dam	R70-3#		Linker		F_pMT_Syp32	R_U8_C_dam	PCR	F_dam	R_dam_Nsi_Flag	Gibson	A21-3
pMT85-SyP32->u8G-dam-flag	SyP32-UTR8-G-dam	R70-3#		Linker		F_pMT_Syp32	R_U8_G_dam	PCR	F_dam	R_dam_Nsi_Flag	Gibson	A20-1
pMT85-SyP32->u8T-dam-flag	SyP32-UTR8-T-dam	R70-3#		Linker		F_pMT_Syp32	R_U8_T_dam	PCR	F_dam	R_dam_Nsi_Flag	Gibson	A19-2

pMT85-SyP32->ATG-dam-flag	SyP32-ATG-dam	R70-3#		Linker		F_pMT_Syp32	R_Syp32_NTG	PCR	F_dam	R_dam_Nsi_Flag	Gibson	A43-1
pMT85-SyP32->CTG-dam-flag	SyP32-CTG-dam	R70-3#		Linker		F_pMT_Syp32	R_Syp32_NTG	PCR	F_dam	R_dam_Nsi_Flag	Gibson	A43-2
pMT85-SyP32->GTG-dam-flag	SyP32-GTG-dam	R70-3#		Linker		F_pMT_Syp32	R_Syp32_NTG	PCR	F_dam	R_dam_Nsi_Flag	Gibson	A43-4
pMT85-SyP32->TTG-dam-flag	SyP32-TTG-dam	R70-3#		Linker		F_pMT_Syp32	R_Syp32_NTG	PCR	F_dam	R_dam_Nsi_Flag	Gibson	A43-6
pMT85-llmp200->ATG-dam-flag	llmp200-ATG-dam	R70-3#		Linker		F_pMT_llmp200	R_mp200_NATG	PCR	F_dam	R_dam_Nsi_Flag	Gibson	A45-5
pMT85-llmp200->CTG-dam-flag	llmp200-CTG-dam	R70-3#		Linker		F_pMT_llmp200	R_mp200_NATG	PCR	F_dam	R_dam_Nsi_Flag	Gibson	A45-1
pMT85-llmp200->GTG-dam-flag	llmp200-GTG-dam	R70-3#		Linker		F_pMT_llmp200	R_mp200_NATG	PCR	F_dam	R_dam_Nsi_Flag	Gibson	A45G-4
pMT85-llmp200->TTG-dam-flag	llmp200-TTG-dam	R70-3#		Linker		F_pMT_llmp200	R_mp200_NATG	PCR	F_dam	R_dam_Nsi_Flag	Gibson	A45T-8
pMT85-SyP32->ATC-ATG-dam-flag	ATC-ATG-dam	R70-3#		Linker		F_pMT_Syp32	R_LL_NTG	PCR	F_dam	R_dam_Nsi_Flag	Gibson	A37-3
pMT85-SyP32->ATC-CTG-dam-flag	ATC-CTG-dam	R70-3#		Linker		F_pMT_Syp32	R_LL_NTG	PCR	F_dam	R_dam_Nsi_Flag	Gibson	A40-2
pMT85-SyP32->ATC-GTG-dam-flag	ATC-GTG-dam	R70-3#		Linker		F_pMT_Syp32	R_LL_NTG	PCR	F_dam	R_dam_Nsi_Flag	Gibson	A37'-1
pMT85-SyP32->ATC-TTG-dam-flag	ATC-TTG-dam	R70-3#		Linker		F_pMT_Syp32	R_LL_NTG	PCR	F_dam	R_dam_Nsi_Flag	Gibson	A37-1

Supplementary Table 2. Tested promoters and 5'-UTRs

Sequences upstream of the Dam ATG are depicted, of known promoters and the promoters and 5'-UTRs selected from the libraries for validation.

Proof of principle

p517	GGCCGCAAAAATTAAATTAAGTTTTCTCCGCTTTAATTAACA ATTTTCTTTTATATAAATAGGATCAAAGATAAAAAAG
p674	GGCCGCCTACTCCAAGAATTATAAGCCTCTCTACAGCTTTAT CTCAAACTTATGTAAAATTAGAGACGTAATTCAAACACG
p036	TCACTTTCAGCAGTTAAAGTTCAGGTGTAAAGTTAACGATTA AGATCAAAAACCGTTCCTAAAAAAAGATCTTTTTCTAAAATCT AAGCGAGTTACAACTCAATTTAAATTTTCTCTCTGGTTGGTT CGCCAACAGTTTTAGCCACTTCAACATTCGTCAAACACCATT AA
p665	TCAGCAATTACAAAAACAAAACAAATAAAAAATAAGGGAATT ACCCCCAAGAAGACCTTTTGTGCTAACGCCAGTTTGGCAAA TCAAGTTCTGATTTTGCAATTATTTTGCTCCATATGAATTACA CTACTCCAAGAATTATAAGCCTCTCTACAGCTTTATCTCAAA CTTATGTAAAATTAGAGACGTAATTCAAACAC

Promoters

p1	ACCCATTGAAGTGGTCGTACTATAGTATAATTCTGATT
p2	ATAATCATCAATGATAAAGCTTTCGTATAATTTATATT
p3	CTCAACACGAGATTTCTTAGAAGTGTATAATATCAATA
p4	GTAAATTCAACATGCTTGCCTGCGTTATAATATAGTTT
p5	TAGGGTCGGTAACCCACTCGCCTCCTATAATCAGTGCA
p6	TTGACCCGATCCATCGCGGTAATTTTATAATCGGACAT
p7	AAAGAGGTGCGTGTCTATACTATTATATAATCTTATCT
p8	CTTTCATATCGCATTAGCATTATATTATAATGAAATTA
p9	GTTGAACTATAGCGTCTCGTTACAGTATAATAATCTAA
p10	TAGCACCTACAATTTAAAGAGTATATATAATTCATATT
p11	TCTTTAAGGTTTTGTATTGGTCATTTATAATCCTCATC
p12	CGCTGTAAGCTAAATTTCAGGGGCCTATAATTTCATGG

5´-UTRs

UTR1	ATAAGAGTGCCTGGATCCAGACAAT
UTR2	ATAGAATTGGGTAAGTAAACTTATC
UTR3	CAACACTGAAGTTTAAGTTGAAACC
UTR4	AGGAGAGATTTTCCAAAGTCCATAT
UTR5	TTAGATATTCGTCTAAGGAGGGATT
UTR6	CGATATTACGTGCTACTTCGACCAG
UTR7	CGCTATTTATTTGACCGCAAGTGTG
UTR8	TCGTTCCACAAATCTACCTGCTTCA

Supplementary Table 3. Dam activity and corresponding Dam copy number of known promoters

* qPCR using oligos that amplify GATC from the genomic 19,580 position. # Estimated from linear regression interpolation using the DNA methylation level. Dam protein copy number / cell was estimated from LC-MSMS (see Methods). In the case of Venus, Western blot cannot be compared. NA: does not apply; ND: not determined. Stdev is the Standard deviation (n=3 in qPCR, n=2 in LC-MSMS).

	**Dam activity (qPCR)***		**Dam abundance**		
**Promoter**	**Average**	**Stdev**	**Copy number per cell**	**Copy number per cell (Stdev)**	**Western blot intensity**
p674-Venus (negative control)	0.010	0.0009	1.2#	0.02	NA
p517	0.41	0.41	11.14	10.29	518.22
p674	0.33	0.14	9.25	3.60	621.71
p036	0.57	0.30	15.24	7.47	2348.49
p665	19.11	7.52	479.86	188.55	9669.73
Syp32 (strong promoter)	3.68	5.17	93.2#	129.65	ND
Il_mp200 (weak promoter)	0.60	0.26	16.08#	6.57	ND

Supplementary Table 4. Correlation between two biological replicas in the transcription and translation screens

Different read count cutoffs (minimal number of reads in one of the two digestions) were applied to find a cutoff (highlighted), which is a compromise (maximise the number of unique sequences and the correlation between the two replicates). The number of filtered reads for each cutoff is indicated. The corresponding unique sequences were determined and the overlap between the 2 replicas and correlation of their DAMRatio was computed. See also Supplementary Figure 2.

Promoter library

	Experiment 1			Experiment 2			Comparison			
**Read count cutoff**	**Filtered DpnI reads**	**Filtered MboI reads**	**Unique sequences**	**Filtered DpnI reads**	**Filtered MboI reads**	**Unique sequences**	**Overlap**	**Combined**	**Pearson r**	**Spearman r**
0	12724070	12207185	2042191	15094952	13026648	1815938	325330	3532799	0.741	0.697
10	11778451	11304930	230930	14224747	12366358	197513	146582	281861	0.869	0.779
20	11249899	10824763	171309	13831620	12068909	155373	103047	223635	0.900	0.807
30	10692050	10334420	136257	13399767	11750024	129575	78863	186969	0.916	0.822
40	10151101	9862285	112617	12951604	11424468	111020	62961	160676	0.926	0.831
50	9633275	9406874	95108	12503919	11099220	96741	51474	140375	0.933	0.838
60	9143314	8981494	81744	12068350	10785510	85489	42899	124334	0.939	0.844
70	8684189	8587479	71247	11648166	10480444	76340	36384	111203	0.943	0.848
80	8258852	8216362	62728	11235969	10182271	68562	31162	100128	0.947	0.852
90	7865006	7865329	55746	10851231	9901233	62127	27128	90745	0.950	0.856
100	7480801	7529615	49706	10480828	9632425	56599	23666	82639	0.953	0.860
110	7129501	7216535	44650	10129045	9372132	51807	20863	75594	0.955	0.862
120	6794114	6930270	40336	9790760	9122177	47625	18451	69510	0.957	0.866
130	6481865	6654402	36570	9459683	8884036	43905	16448	64027	0.959	0.869
140	6198276	6395194	33369	9154983	8652495	40643	14710	59302	0.960	0.870
150	5926120	6159868	30568	8861348	8436842	37773	13267	55074	0.961	0.871

160	5666603	5921756	28004	8576404	8229177	35163	11979	51188	0.962	0.873
170	5440445	5707603	25864	8310125	8031184	32854	10812	47906	0.964	0.876
180	5213640	5497533	23873	8047270	7831554	30683	9803	44753	0.965	0.880
190	4996040	5296303	22060	7795391	7655584	28785	8933	41912	0.966	0.883
200	4797877	5111891	20489	7556551	7474994	27014	8192	39311	0.967	0.885
300	3261917	3663381	10597	5698651	6011028	15752	3788	22561	0.973	0.899
400	2321529	2732745	6209	4415370	4953042	10107	2075	14241	0.978	0.902
500	1703209	2110624	3939	3521193	4191960	7009	1274	9674	0.980	0.915
600	1273293	1666291	2636	2883943	3597731	5104	817	6923	0.983	0.919
700	978608	1349324	1859	2355936	3119712	3794	553	5100	0.984	0.921
800	756633	1102551	1341	1961356	2737551	2916	392	3865	0.984	0.926
900	596219	886870	970	1650629	2429350	2298	268	3000	0.985	0.949
1000	463623	731809	715	1433309	2167365	1866	200	2381	0.985	0.951

5'-UTR library with strong promoter

	Experiment 1			Experiment 2			Comparison			
**Read count cutoff**	**Filtered DpnI reads**	**Filtered MboI reads**	**Unique sequences**	**Filtered DpnI reads**	**Filtered MboI reads**	**Unique sequences**	**Overlap**	**Combined**	**Pearson r**	**Spearman r**
0	16620627	13616606	2925359	8568411	19027119	2054085	434517	4544927	0.718	0.729
10	14908604	11850781	401155	8017650	17705195	289539	141022	549672	0.882	0.871
20	13846497	10605555	252014	7619909	16961261	206631	85361	373284	0.909	0.896
30	12913566	9602381	181670	7234755	16256432	162174	59529	284315	0.922	0.908
40	12097603	8782223	140089	6874740	15592403	133071	44779	228381	0.931	0.915
50	11376578	8093893	112512	6529186	14963310	111715	34985	189242	0.938	0.920
60	10733482	7510828	92940	6207070	14375294	95518	28219	160239	0.943	0.924
70	10171210	7005293	78594	5912550	13825851	82862	23263	138193	0.947	0.927
80	9668858	6564590	67607	5645984	13309025	72735	19603	120739	0.951	0.930

90	9212140	6168757	58872	5398926	12837521	64528	16858	106542	0.954	0.932
100	8787157	5815458	51746	5166061	12408647	57782	14582	94946	0.957	0.934
110	8398188	5496473	45869	4951144	11992537	51971	12816	85024	0.959	0.936
120	8049962	5215959	41114	4756722	11617798	47213	11291	77036	0.961	0.938
130	7727352	4956141	37056	4573559	11258920	43048	10068	70036	0.962	0.939
140	7413977	4711126	33453	4405130	10922402	39444	9071	63826	0.964	0.941
150	7137699	4490608	30480	4243633	10606061	36283	8172	58591	0.965	0.943
160	6877981	4287868	27884	4094745	10308097	33515	7406	53993	0.966	0.944
170	6630998	4099795	25582	3956270	10032414	31103	6775	49910	0.967	0.945
180	6396955	3922632	23536	3826340	9776679	28992	6219	46309	0.968	0.946
190	6184783	3758747	21775	3707848	9514012	27022	5723	43074	0.969	0.948
200	5973647	3606444	20149	3593305	9272348	25283	5273	40159	0.970	0.948
300	4439082	2509081	10670	2700449	7380248	14348	2723	22295	0.974	0.954
400	3446911	1852590	6482	2150029	6102220	9288	1561	14209	0.980	0.957
500	2731938	1422287	4229	1744381	5178878	6445	987	9687	0.981	0.961
600	2244079	1111559	2947	1447557	4454398	4666	676	6937	0.981	0.960
700	1876923	886641	2146	1234400	3903811	3544	481	5209	0.984	0.966
800	1606884	723720	1635	1053790	3454387	2740	349	4026	0.984	0.966
900	1354354	573739	1218	931906	3097085	2206	264	3160	0.985	0.960
1000	1169641	472746	947	821906	2754951	1751	204	2494	0.985	0.956

5'-UTR library with weak promoter

	Experiment 1			Experiment 2			Comparison			
**Read count cutoff**	**Filtered DpnI reads**	**Filtered MboI reads**	**Unique sequences**	**Filtered DpnI reads**	**Filtered MboI reads**	**Unique sequences**	**Overlap**	**Combined**	**Pearson r**	**Spearman r**
0	18412532	16217588	2961856	10737660	11729822	1474115	471982	3963989	0.458	0.410
10	16578951	14649482	353733	9830249	10840867	257918	187241	424410	0.670	0.617

20	15406303	13658832	238785	8997928	10062944	171484	114392	295877	0.761	0.694
30	14302147	12758190	179773	8194137	9323172	125503	78981	226295	0.809	0.737
40	13286823	11941401	142213	7481322	8656137	96901	58016	181098	0.838	0.764
50	12360592	11206038	116024	6857920	8074055	77714	44588	149150	0.859	0.785
60	11527051	10548857	96959	6297853	7549151	63703	35096	125566	0.873	0.801
70	10769301	9949475	82295	5807768	7082929	53304	28328	107271	0.883	0.815
80	10072809	9402742	70685	5379216	6671604	45405	23333	92757	0.892	0.829
90	9447735	8908829	61440	4993682	6297870	39081	19493	81028	0.901	0.841
100	8872448	8458923	53886	4655528	5968535	34121	16529	71478	0.906	0.851
110	8349858	8049773	47658	4346708	5655852	29914	14230	63342	0.912	0.860
120	7878888	7671495	42481	4064881	5371870	26437	12245	56673	0.916	0.868
130	7441467	7325514	38098	3815773	5115817	23577	10695	50980	0.921	0.874
140	7032131	6998669	34282	3581260	4874730	21070	9362	45990	0.926	0.879
150	6660823	6706815	31071	3375208	4655404	18984	8286	41769	0.930	0.882
160	6329378	6438692	28357	3189964	4456106	17207	7368	38196	0.931	0.885
170	6016156	6183898	25955	3018918	4269299	15658	6643	34970	0.934	0.890
180	5712436	5943526	23768	2862907	4096361	14308	5983	32093	0.937	0.896
190	5436624	5715876	21868	2714243	3933358	13106	5410	29564	0.939	0.897
200	5171049	5493073	20109	2570576	3781735	12024	4917	27216	0.941	0.901
300	3293093	3879122	9916	1631622	2656833	5875	2155	13636	0.958	0.927
400	2239449	2892947	5684	1150694	2019184	3482	1176	7990	0.965	0.936
500	1567348	2235887	3523	831714	1583555	2225	697	5051	0.966	0.935
600	1133579	1786522	2339	639112	1261829	1510	449	3400	0.970	0.944
700	849801	1466732	1650	495256	1046290	1088	295	2443	0.971	0.946
800	652739	1220563	1203	388828	872690	801	191	1813	0.971	0.953
900	506287	1026225	896	319263	757721	630	139	1387	0.975	0.953
1000	387479	879172	685	259604	638428	481	102	1064	0.972	0.958

Supplementary Table 5. DAMRatio, protein copy number and mRNA level of individual clones

Validation of 12 promoters and eight 5´-UTRs randomly picked from the screenings. Dam Activity (qPCR with 4xGATC oligos), Dam quantification by Proteomics (a), and mRNA by RT-qPCR (b) are shown. "Screening data" are the values form the screenings. "Validation data" correspond to individual clones. Estimated copy number from DAMRatio is calculated from Experiment 1. MPN001 is used for normalization in LC-MSMS, 16S in the Dam activity assay, and MPN517 is used for RT-qPCR (oligo set 1=qdam, oligo set 2=qdam2). NA= Does not apply. Stdev is the Standard deviation (n=3 for qPCR, n=24 for the copy number estimation.

a. Dam activity and protein copy number

	Screening data							Validation data				
	**Experiment 1**			**Experiment 2**				**Dam activity**		**Proteomics**		
**Promoter Set 1**	**DpnI read count**	**MboI read count**	**DAMRatio**	**DpnI read count**	**MboI read count**	**DAMRatio**	**Estimated copy number from DAMRatio**	**MboI/DpnI ratio (qPCR)**	**MboI/DpnI ratio Stdev**	**LC-MSMS**	**Copy number per cell**	**Copy number per cell (Stdev)**
p6	0	237	2.907	2	68	2.004	903.21	7.74	1.48	0.619	757.70	40.75
p7	2	144	2.215	6	92	1.765	180.70	10.78	3.87	0.225	173.13	6.19
p8	3	144	2.090	0	44	2.295	135.16	32.12	4.00	0.219	150.43	5.28
p9	21	514	1.900	45	1105	2.023	86.92	56.79	14.01	0.195	130.71	4.53
p10	15	212	1.655	32	211	1.450	49.17	33.69	12.05	0.105	41.01	1.67
p11	63	121	0.811	6	128	1.907	6.91	2.46	0.62	0.024	6.16	0.43
p12	242	92	0.113	28	86	1.119	1.37	0.01	0.002	0.000	NA	NA

	**Experiment 1**			**Experiment 2**				**Dam activity**		**Proteomics**		
**Promoter Set 2**	**DpnI read count**	**MboI read count**	**DAMRatio**	**DpnI read count**	**MboI read count**	**DAMRatio**	**Estimated copy number from DAMRatio**	**MboI/DpnI ratio (qPCR)**	**MboI/DpnI ratio Stdev**	**LC-MSMS**	**Copy number per cell**	**Copy number per cell (Stdev)**
p1	0	93	2.503	1	58	2.112	1149.52	0.17	0.00	1.118	2334.84	174.16
p2	2	91	2.017	1	38	1.932	279.50	1.69	1.96	0.152	105.86	3.658
p3	44	96	0.864	57	126	0.982	9.79	0.08	0.02	0.035	12.00	0.701
p4	92	37	0.142	30	70	1.002	1.20	0.014	0.0013	0.009	1.27	0.126
p5	91	7	-0.530	117	94	0.548	0.17	0.007	0.0019	0	NA	NA

	**Experiment 1**			**Experiment 2**				**Dam activity**		**Proteomics**		
**5´-UTR Set**	**DpnI read count**	**MboI read count**	**DAMRatio**	**DpnI read count**	**MboI read count**	**DAMRatio**	**Estimated copy number from DAMRatio**	**MboI/DpnI ratio (qPCR)**	**MboI/DpnI ratio Stdev**	**LC-MSMS**	**Copy number per cell**	**Copy number per cell (Stdev)**
UTR1	0	149	3.350	17	255	1.893	2521.74	41.015	10.62	0.542	658.294	33.88
UTR2	4	114	2.535	2	70	2.115	462.93	18.324	2.02	0.682	935.668	53.72
UTR3	7	145	2.435	12	162	1.839	375.57	18.368	3.98	0.640	784.816	42.68
UTR4	19	249	2.270	1	17	1.695	266.76	32.944	1.92	1.033	1642.460	62.51
UTR5	87	684	2.065	26	320	1.816	173.85	5.742	0.74	0.117	62.506	2.29
UTR6	152	247	1.383	55	119	1.072	42.09	2.318	0.86	0.056	19.619	0.99
UTR7	138	17	0.286	18	102	1.475	4.29	0.031	0.01	0.018	3.770	0.30
UTR8	364	23	-0.009	11	251	2.063	2.32	0.012	0.00	0	NA	NA

b. mRNA level

	Validation data			
	**RT-qPCR dam**		**RT-qPCR dam 2**	
**Promoter set 1**	**Average**	**Stdev**	**Average**	**Stdev**
p6	4.963	0.65	23.482	2.30
p7	2.198	0.46	14.673	2.60
p8	1.960	0.10	10.497	0.65
p9	2.937	1.25	12.645	2.09
p10	1.016	0.08	7.144	0.85
p11	0.846	0.12	6.526	0.51
p12	0.227	0.03	2.887	1.05

	**qPCR dam**		**qPCR dam 2**	
**Promoter set 2**	**Average**	**Stdev**	**Average**	**Stdev**
p1	11.679	5.27	41.903	4.62
p2	3.556	0.31	15.192	0.94
p3	1.065	0.24	5.739	0.24
p4	0.616	0.19	3.437	0.16
p5	0.202	0.04	2.109	0.18

	**qPCR dam**		**qPCR dam 2**	
**5´-UTR set**	**Average**	**Stdev**	**Average**	**Stdev**
UTR1	26.498	10.96	30.097	7.04
UTR2	14.532	3.40	18.545	2.26
UTR3	9.100	2.31	14.156	2.62
UTR4	14.989	1.17	18.009	3.81
UTR5	4.695	0.21	17.602	0.32
UTR6	2.172	0.33	5.134	0.49
UTR7	2.437	0.29	5.404	0.29
UTR8	0.150	0.05	0.657	0.14

Supplementary Table 6. Estimated Dam protein copy number per cell from LC-MSMS data

Dam protein copy number was estimated from a regression model using proteome-wide copy number data derived from LC-MSMS. We used 24 regression models from all Proteomics experiments and combined the averages. Stdev is the standard deviation (n=24). NA=not applicable.

	Constructs for proof of principle				Individual promoter constructs											
	**p517**	**p674**	**p036**	**p665**	**p1**	**p2**	**p3**	**p4**	**p5**	**p6**	**p7**	**p8**	**p9**	**p10**	**p11**	**p12**
Estimation 1	3.22	4.27	26.46	454.17	2203.37	100.42	11.40	1.21	NA	716.73	164.14	142.64	123.96	38.93	5.85	NA
Estimation 2	3.01	4.01	25.78	468.33	2345.20	100.47	10.92	1.10	NA	745.88	165.84	143.71	124.54	38.22	5.53	NA
Estimation 3	3.14	4.18	26.81	484.08	2415.62	104.19	11.38	1.16	NA	770.19	171.80	148.93	129.10	39.72	5.77	NA
Estimation 4	3.26	4.35	27.92	506.07	2531.01	108.69	11.83	1.20	NA	805.69	179.35	155.44	134.71	41.38	6.00	NA
Estimation 5	3.49	4.62	28.34	478.30	2298.87	106.70	12.27	1.32	NA	752.78	173.90	151.25	131.55	41.60	6.32	NA
Estimation 6	3.63	4.78	28.43	457.00	2137.76	104.63	12.49	1.39	NA	713.65	169.09	147.42	128.53	41.46	6.51	NA
Estimation 7	3.37	4.45	27.07	450.97	2152.19	101.29	11.76	1.27	NA	708.31	164.72	143.35	124.76	39.66	6.08	NA
Estimation 8	3.61	4.76	28.89	478.48	2275.96	107.81	12.57	1.37	NA	750.80	175.14	152.47	132.73	42.29	6.51	NA
Estimation 9	3.14	4.18	27.09	497.79	2508.46	106.15	11.43	1.15	NA	794.24	175.56	152.05	131.69	40.22	5.78	NA
Estimation 10	3.35	4.42	27.08	455.72	2186.71	101.83	11.73	1.26	NA	716.90	165.87	144.29	125.51	39.74	6.05	NA
Estimation 11	3.69	4.86	28.83	461.83	2156.40	105.92	12.67	1.42	NA	720.81	171.08	149.18	130.08	42.02	6.61	NA
Estimation 12	3.53	4.67	28.74	486.91	2345.32	108.40	12.43	1.33	NA	766.82	176.79	153.73	133.68	42.21	6.40	NA
Estimation 13	3.19	4.23	26.46	460.84	2254.21	101.10	11.35	1.19	NA	728.99	165.67	143.87	124.93	39.00	5.80	NA
Estimation 14	3.37	4.46	27.41	464.59	2238.33	103.41	11.85	1.27	NA	731.71	168.66	146.66	127.53	40.26	6.10	NA
Estimation 15	3.33	4.40	26.87	449.39	2149.20	100.73	11.66	1.26	NA	706.26	163.92	142.63	124.11	39.39	6.02	NA
Estimation 16	3.55	4.69	28.61	479.09	2292.88	107.32	12.41	1.34	NA	753.10	174.67	151.98	132.23	41.95	6.41	NA
Estimation 17	3.32	4.41	27.78	488.49	2402.32	106.62	11.88	1.23	NA	773.93	175.00	151.90	131.84	41.00	6.06	NA
Estimation 18	3.86	5.09	30.39	492.04	2311.21	112.21	13.32	1.48	NA	769.28	181.57	158.24	137.91	44.35	6.93	NA
Estimation 19	3.32	4.40	27.62	482.72	2365.83	105.70	11.83	1.23	NA	764.03	173.32	150.48	130.65	40.73	6.05	NA
Estimation 20	2.91	3.92	27.15	553.00	2950.81	111.64	11.11	1.03	NA	897.04	187.97	161.97	139.57	40.88	5.48	NA
Estimation 21	3.10	4.13	26.86	496.12	2507.01	105.51	11.32	1.13	NA	792.21	174.66	151.23	130.95	39.92	5.71	NA
Estimation 22	4.18	5.48	31.47	479.87	2179.95	112.99	14.04	1.63	NA	743.05	180.94	158.16	138.25	45.57	7.41	NA
Estimation 23	3.51	4.65	28.95	500.10	2434.98	110.20	12.45	1.31	NA	790.04	180.32	156.65	136.09	42.63	6.38	NA
Estimation 24	3.35	4.44	27.89	487.93	2392.52	106.79	11.94	1.24	NA	772.38	175.13	152.05	132.01	41.14	6.10	NA
Average	**3.39**	**4.49**	**27.87**	**479.74**	**2334.84**	**105.86**	**12.00**	**1.27**	NA	**757.70**	**173.13**	**150.43**	**130.71**	**41.01**	**6.16**	NA
Stdev	0.275	0.340	1.266	22.344	174.155	3.658	0.701	0.126	NA	40.753	6.188	5.278	4.533	1.666	0.432	NA

	Individual 5'-UTR constructs with strong promoter							
	**UTR1**	**UTR2**	**UTR3**	**UTR4**	**UTR5**	**UTR6**	**UTR7**	**UTR8**
Estimation 1	622.86	884.72	742.33	1551.22	59.32	18.63	3.58	NA
Estimation 2	646.37	924.59	773.06	1639.49	58.72	18.02	3.35	NA
Estimation 3	667.64	954.28	798.20	1690.03	60.97	18.76	3.50	NA
Estimation 4	698.28	998.57	835.03	1769.87	63.56	19.52	3.63	NA
Estimation 5	654.73	928.06	779.50	1621.81	63.23	19.99	3.88	NA
Estimation 6	622.19	876.64	738.54	1517.26	62.56	20.18	4.03	NA
Estimation 7	616.45	872.42	733.35	1520.73	60.16	19.12	3.74	NA
Estimation 8	653.62	924.34	777.28	1609.36	64.11	20.43	4.01	NA
Estimation 9	687.89	985.37	823.31	1751.17	61.91	18.91	3.49	NA
Estimation 10	623.61	883.63	742.32	1543.25	60.37	19.11	3.72	NA
Estimation 11	628.54	885.23	745.92	1531.12	63.37	20.47	4.09	NA
Estimation 12	666.81	945.64	794.08	1653.79	64.19	20.27	3.92	NA
Estimation 13	633.05	900.84	755.17	1584.11	59.56	18.59	3.54	NA
Estimation 14	636.27	902.37	757.73	1578.26	61.23	19.33	3.74	NA
Estimation 15	614.54	870.13	731.26	1517.89	59.79	18.98	3.70	NA
Estimation 16	655.25	927.92	779.76	1619.12	63.68	20.20	3.94	NA
Estimation 17	671.75	957.06	801.81	1686.17	62.69	19.50	3.69	NA
Estimation 18	670.45	945.51	796.19	1638.86	67.00	21.55	4.28	NA
Estimation 19	663.36	944.39	791.50	1661.83	62.23	19.40	3.69	NA
Estimation 20	772.98	1121.43	931.05	2033.94	63.89	18.71	3.26	NA
Estimation 21	685.97	983.22	821.25	1749.07	61.49	18.74	3.45	NA
Estimation 22	649.52	909.21	768.47	1557.31	68.22	22.48	4.63	NA
Estimation 23	686.34	975.68	818.32	1712.89	65.02	20.37	3.90	NA
Estimation 24	670.59	954.77	800.16	1680.39	62.86	19.59	3.72	NA
Average	**658.29**	**935.67**	**784.82**	**1642.46**	**62.51**	**19.62**	**3.77**	NA
Stdev	33.877	53.719	42.677	111.279	2.295	0.991	0.298	NA

Supplementary Table 7. Growth phenotype of individual clones

Growth curves were performed by measuring the pH indicator colour change (see Methods). "Late time slope" equals the maximum slope of the pH color change. "Early time slope " equals the maximum slope of the pH color change in the first 24 hours. Dam protein copy number per cell was estimated from LC-MSMS (see Supplementary Data 2 and Methods). NC: negative control.

	**Late time slope**	**Early time slope**	**Dam copy number per cell**	**Description**
Empty vector	0.103	0.0018	NA	NC
p665-Venus	0.103	0.0014	NA	NC
p517	0.092	0.0010	3.39	Proof of principle
p674	0.094	0.0011	4.49	Proof of principle
p036	0.094	0.0013	27.87	Proof of principle
p665	0.081	0.0011	479.74	Proof of principle
p12	0.090	0.0009	1.37	Promoter validation set 1
p11	0.084	0.0007	6.16	Promoter validation set 1
p10	0.084	0.0030	41.01	Promoter validation set 1
p9	0.082	0.0007	130.71	Promoter validation set 1
p8	0.082	0.0009	150.43	Promoter validation set 1
p7	0.082	0.0009	173.13	Promoter validation set 1
p6	0.083	0.0007	757.70	Promoter validation set 1
p5	0.096	0.0008	0.17	Promoter validation set 2
p4	0.093	0.0009	1.27	Promoter validation set 2
p3	0.095	0.0008	12.00	Promoter validation set 2
p2	0.095	0.0010	105.86	Promoter validation set 2
p1	0.077	0.0009	2334.84	Promoter validation set 2

Supplementary Table 8. Odd-ratio cancels nucleotide biases

Odd ratio is the nucleotide frequency between high-productive and low-productive promoter sequences. This table is similar to the Odd ratio of promoters in Fig. 3, but sequences were randomized. As it is shown here (the table is pseudocoloured) we cannot observe the nucleotide bias from randomly selected sequences. Randomly selected sequences do not overlap with each other.

a. Randomly selected high-productive promoters (n=35,417) and low-productive promoters (n=47,222)

	-37	-36	-35	-34	-33	-32	-31	-30	-29	-28	-27	-26	-25	-24	-23	-22	-21	-20	-19	-18	-17	-16	-15	-14	-13	**T**	**A**	**T**	**A**	**A**	**T**	-6	-5	-4	-3	-2	-1	+1
A	0.0	0.0	0.0	0.0	0.0	0.0	0.0	0.0	0.0	0.0	0.0	0.0	0.0	0.0	0.0	0.0	0.0	0.0	0.0	0.0	0.0	0.0	0.0	0.0	0.0							0.0	0.0	0.0	0.0	0.0	0.0	0.0
C	0.0	0.0	0.0	0.0	0.0	0.0	0.0	0.0	0.0	0.0	0.0	0.0	0.0	0.0	0.0	0.0	0.0	0.0	0.0	0.0	0.0	0.0	0.0	0.0	0.0							0.0	0.1	0.0	0.0	0.0	0.0	0.0
G	0.0	0.0	0.0	0.0	0.0	0.0	0.0	0.0	0.0	0.0	0.0	0.0	0.0	0.0	0.0	0.0	0.0	0.0	0.0	0.0	0.0	0.0	0.0	0.0	0.0							0.0	0.0	0.0	0.0	0.0	0.0	0.0
T	0.0	0.0	0.0	0.0	0.0	0.0	0.0	0.0	0.0	0.0	0.0	0.0	0.0	0.0	0.0	0.0	0.0	0.0	0.0	0.0	0.0	0.0	0.0	0.0	0.0							0.0	0.0	0.0	0.0	0.0	0.0	0.0

b. Randomly selected high-productive 5'-UTRs (n=55,329) and low-productive 5'-UTRs (n=39,616)

	-25	-24	-23	-22	-21	-20	-19	-18	-17	-16	-15	-14	-13	-12	-11	-10	-9	-8	-7	-6	-5	-4	-3	-2	-1
A	0.0	0.0	0.0	0.0	0.0	0.0	0.0	0.0	0.0	0.0	0.0	0.0	0.0	0.0	0.0	0.0	0.0	0.0	0.0	0.0	0.0	0.0	0.0	0.0	0.0
C	0.0	0.0	0.0	0.0	0.0	0.0	0.0	0.0	0.0	0.0	0.0	0.0	0.1	0.0	0.0	0.0	0.0	0.0	0.0	0.0	0.0	0.0	0.0	0.0	0.0
G	0.0	0.0	0.0	0.0	0.0	0.0	0.0	0.0	0.1	0.0	0.0	0.0	0.0	0.0	0.0	0.0	0.0	0.0	0.0	0.0	0.0	0.0	0.0	0.0	0.0
T	0.0	0.0	0.0	0.0	0.0	0.0	0.0	0.0	0.0	0.0	0.0	0.0	0.0	0.0	0.0	0.0	0.0	0.0	0.0	0.0	0.0	0.0	0.0	0.0	0.0

c. Example of the background frequency and how to calculate log2ratio for promoter library

Promoter (cutoff=0;e xp1) - log2 odd-ratio (background bias) against uniform 40% GC bias

	-37	-36	-35	-34	-33	-32	-31	-30	-29	-28	-27	-26	-25	-24	-23	-22	-21	-20	-19	-18	-17	-16	-15	-14	-13	**T**	**A**	**T**	**A**	**A**	**T**	-6	-5	-4	-3	-2	-1	+1
A	-0.1	-0.1	0.1	-0.1	-0.1	-0.1	-0.1	-0.1	-0.1	-0.1	0.0	0.0	-0.1	-0.1	0.0	-0.1	-0.1	-0.1	-0.1	-0.1	-0.1	-0.1	-0.1	0.3	-0.1							0.2	-0.2	0.0	0.0	0.1	-0.1	-0.1
C	0.2	0.0	-0.1	-0.1	-0.1	-0.2	-0.2	-0.2	-0.1	-0.2	-0.1	-0.1	-0.1	-0.2	-0.2	-0.2	-0.2	-0.2	-0.2	-0.2	-0.1	-0.1	-0.1	-0.2	-0.2							-0.1	0.1	0.1	-0.1	-0.2	-0.2	-0.2
G	-0.2	0.0	0.0	-0.1	0.0	0.1	0.1	0.0	0.0	0.0	0.0	0.0	0.0	0.0	0.0	0.0	0.0	0.0	0.0	0.0	0.0	0.0	0.0	-0.2	0.0							-0.1	-0.1	-0.2	-0.2	0.0	0.0	0.0
T	0.1	0.1	0.0	0.2	0.2	0.2	0.2	0.1	0.1	0.1	0.1	0.1	0.2	0.2	0.1	0.2	0.2	0.2	0.2	0.2	0.1	0.2	0.1	-0.1	0.2							-0.1	0.1	0.1	0.2	0.1	0.2	0.2

Promoter (cutoff=50; exp1) - log2 odd-ratio (background bias) against uniform 40% GC bias

	-37	-36	-35	-34	-33	-32	-31	-30	-29	-28	-27	-26	-25	-24	-23	-22	-21	-20	-19	-18	-17	-16	-15	-14	-13	**T**	**A**	**T**	**A**	**A**	**T**	-6	-5	-4	-3	-2	-1	+1
A	-0.1	-0.1	0.1	-0.1	-0.1	-0.1	-0.1	-0.1	-0.1	-0.1	0.0	0.0	-0.1	-0.1	0.0	-0.1	-0.1	-0.1	-0.1	0.0	-0.1	-0.1	-0.1	0.3	-0.1							0.2	-0.2	0.0	0.0	0.1	-0.1	-0.1
C	0.2	0.0	-0.1	-0.1	-0.1	-0.2	-0.2	-0.2	-0.1	-0.2	-0.1	-0.2	-0.1	-0.2	-0.2	-0.2	-0.2	-0.2	-0.2	-0.2	-0.1	-0.1	-0.1	-0.2	-0.2							-0.1	0.1	0.1	-0.1	-0.2	-0.3	-0.2
G	-0.2	0.0	0.1	-0.1	0.0	0.1	0.1	0.0	0.0	0.0	0.0	0.0	0.0	0.0	0.0	0.0	0.0	0.0	0.0	0.0	0.0	0.0	0.0	-0.2	0.0							-0.1	-0.1	-0.2	-0.2	0.0	0.0	0.0
T	0.1	0.1	0.0	0.2	0.2	0.2	0.2	0.1	0.1	0.1	0.1	0.1	0.2	0.2	0.1	0.2	0.2	0.2	0.2	0.2	0.1	0.2	0.2	-0.1	0.2							-0.1	0.1	0.1	0.2	0.1	0.2	0.2

Promoter (cutoff=50; exp1) - high productive promoter - log2odd ratio against uniform 40% GC bias

	-37	-36	-35	-34	-33	-32	-31	-30	-29	-28	-27	-26	-25	-24	-23	-22	-21	-20	-19	-18	-17	-16	-15	-14	-13	**T**	**A**	**T**	**A**	**A**	**T**	-6	-5	-4	-3	-2	-1	+1
A	-0.2	-0.2	0.0	-0.1	-0.1	-0.1	0.0	0.0	0.0	0.0	-0.1	0.0	-0.1	-0.1	0.0	0.0	0.1	-0.1	0.0	0.1	-0.1	0.3	0.1	0.3	-0.3							0.3	0.0	0.2	0.1	0.3	0.1	-0.3
C	0.3	0.0	-0.1	0.0	0.0	-0.1	-0.2	-0.3	-0.2	-0.3	-0.2	-0.2	-0.1	-0.1	-0.2	-0.3	-0.3	-0.3	-0.4	-0.6	-0.5	-0.8	-1.1	-1.8	-2.2							-0.3	-0.3	-0.3	-0.3	-0.6	-0.5	-0.1
G	-0.3	-0.1	0.1	0.0	0.0	-0.1	-0.1	-0.1	-0.2	-0.1	0.0	0.0	0.0	0.0	0.0	0.0	-0.3	-0.1	-0.2	-0.2	-0.5	-0.1	-1.0	0.0	0.4							-0.5	-0.1	-0.4	-0.3	-0.1	-0.1	-0.2
T	0.2	0.2	0.0	0.1	0.1	0.2	0.2	0.2	0.2	0.2	0.2	0.1	0.1	0.1	0.1	0.2	0.3	0.3	0.3	0.3	0.6	0.1	0.7	0.3	0.5							0.1	0.3	0.2	0.2	0.1	0.2	0.4

Promoter (cutoff=50; exp1) - low productive promoter - log2odd ratio against uniform 40% GC bias

	-37	-36	-35	-34	-33	-32	-31	-30	-29	-28	-27	-26	-25	-24	-23	-22	-21	-20	-19	-18	-17	-16	-15	-14	-13	**T**	**A**	**T**	**A**	**A**	**T**	-6	-5	-4	-3	-2	-1	+1
A	-0.1	-0.1	0.1	-0.1	-0.1	-0.2	-0.2	-0.1	-0.1	-0.1	0.0	0.0	-0.1	-0.1	0.0	-0.1	-0.2	-0.1	-0.2	-0.2	-0.1	-0.4	-0.2	0.2	0.0							0.1	-0.3	-0.2	-0.1	-0.1	-0.1	0.1
C	0.2	0.0	-0.1	-0.1	-0.2	-0.2	-0.2	-0.1	0.0	-0.1	-0.1	-0.1	-0.2	-0.2	-0.2	-0.2	-0.2	-0.1	0.0	0.0	0.1	0.2	0.3	0.3	0.4							0.1	0.4	0.3	0.0	0.0	-0.1	-0.3
G	-0.1	0.0	0.0	-0.1	-0.1	0.1	0.1	0.1	0.1	0.1	0.0	0.0	0.0	0.0	0.0	0.0	0.2	0.1	0.1	0.1	0.3	0.0	0.4	-0.3	-0.4							0.1	-0.1	-0.1	-0.1	0.0	0.1	0.1
T	0.0	0.0	0.0	0.2	0.2	0.2	0.2	0.1	0.1	0.1	0.1	0.1	0.2	0.2	0.2	0.2	0.1	0.1	0.1	0.1	-0.2	0.2	-0.3	-0.4	0.0							-0.3	0.0	0.0	0.1	0.1	0.1	0.0

Promoter (cutoff=50; exp1) - log2 odd-ratio between high and low productive promoter

	-37	-36	-35	-34	-33	-32	-31	-30	-29	-28	-27	-26	-25	-24	-23	-22	-21	-20	-19	-18	-17	-16	-15	-14	-13	**T**	**A**	**T**	**A**	**A**	**T**	-6	-5	-4	-3	-2	-1	1
A	-0.1	-0.1	0.0	0.0	0.0	0.1	0.2	0.2	0.1	0.1	0.0	0.0	0.0	0.0	0.0	0.1	0.2	0.0	0.2	0.3	-0.1	0.8	0.3	0.1	-0.2							0.2	0.3	0.3	0.2	0.3	0.2	-0.4
C	0.1	0.0	0.1	0.1	0.1	0.1	0.0	-0.2	-0.2	-0.2	-0.1	0.0	0.1	0.1	0.0	-0.1	-0.2	-0.2	-0.4	-0.6	-0.6	-1.0	-1.4	-2.1	-2.5							-0.3	-0.7	-0.6	-0.4	-0.5	-0.3	0.2
G	-0.2	-0.1	0.1	0.1	0.0	-0.2	-0.2	-0.2	-0.3	-0.2	-0.1	0.0	0.1	0.1	0.1	-0.1	-0.4	-0.2	-0.3	-0.3	-0.8	-0.1	-1.3	0.2	0.8							-0.6	0.0	-0.3	-0.2	-0.1	-0.2	-0.3
T	0.1	0.1	0.0	-0.1	-0.1	0.0	0.0	0.1	0.2	0.1	0.1	0.0	-0.1	-0.1	0.0	0.0	0.1	0.2	0.1	0.2	0.8	-0.1	1.0	0.7	0.6							0.4	0.2	0.2	0.1	0.0	0.1	0.3

Supplementary Table 9. Promoter motifs found by EXTREME program

Discovered sequence motifs (gapped kmers) in productive sequences using EXTREME - a motif discovery algorithm designed for high-throughput sequencing data.

a. Motifs detected in promoter study

Motif in promoter	# of cases found in high-productive sequences	# of cases found in low-productive sequences	Corrected Z-score	Z-score
ATAAAT	2439	1469	5.37	25.31
TATAAA	3931	2637	5.34	25.20
TAGNATA	1826	1002	5.22	26.03
ATTNATA	2405	1427	5.19	25.89
TATNAAA	2342	1390	5.12	25.53
AATNNATA	2846	1704	5.37	27.67
ATTNNNTAA	2778	1409	7.11	36.47
TATNNNAAA	3586	2250	5.49	28.17
ATTNNNAAT	1555	780	5.41	27.75
TATNNNATA	3191	1981	5.30	27.19
ATTNNNNAAT	1430	548	7.90	37.68
TATNNNNTAA	4278	2528	7.30	34.81
TTANNNNAAA	2275	1301	5.66	27.00
AATNNNNAAT	2516	1511	5.42	25.85
AATNNNNNAAT	2117	1117	7.07	29.92
ATTNNNNNAAA	2034	1125	6.41	27.10
ATTNNNNNATA	3752	2464	6.14	25.95
ATTNNNNNNTAA	2742	1918	5.06	18.81
AATNNNNNNNNNAAA	1357	877	5.76	16.21
AATNNNNNNNNNNAAA	1199	765	5.55	15.69

b. Motifs detected in 5'-UTR study with strong promoter

No motif detected in EXTREME

c. Motifs detected in 5'-UTR study with weak promoter

Motif in 5'-UTR	# of cases found in high-productive sequences	# of cases found in low-productive sequences	Corrected Z-score	Z-score
AATTAT	816	314	5.48	28.33
AATATA	943	389	5.43	28.09
AAANTAT	863	337	5.75	28.65
AATNATA	783	308	5.43	27.07
AAANAAT	909	401	5.09	25.37
AAANATA	922	411	5.06	25.21
AAANTAA	884	389	5.03	25.10
AAANNATT	847	335	5.75	27.97
AAANNATA	887	362	5.67	27.59

TAANNATA	757	298	5.46	26.59
AAANNAAA	1032	467	5.37	26.15
AATNNTAA	750	303	5.28	25.68
AAANNTAT	757	312	5.18	25.19
AAANNAAT	832	360	5.11	24.88
AATNNATA	668	265	5.09	24.76
AAANNNATA	831	320	6.08	28.57
TAANNNATA	708	271	5.65	26.55
AAANNNTTA	820	335	5.64	26.50
TAANNNTAA	728	289	5.50	25.82
AAANNNAAT	797	350	5.09	23.89
AAANNNTAA	817	363	5.07	23.83
AAANNNATT	768	336	5.02	23.57
AAANNNNATA	767	327	5.40	24.33
AAANNNNTAT	758	322	5.40	24.30
ATANNNNTAA	642	263	5.19	23.37
TAANNNNATA	657	273	5.16	23.24
AAANNNNAAT	744	327	5.12	23.06
AAANNNNNATA	725	291	5.84	25.44
ATANNNNNTTA	632	249	5.57	24.27
AAANNNNNNATA	657	278	5.39	22.73
TAANNNNNNATA	564	229	5.25	22.14
ATANNNNNNTTA	560	236	5.00	21.09
ATANNNNNNNATA	542	231	5.02	20.46
TAANNNNNNNNATA	489	201	5.14	20.31
AATNNNNNNNNNAAT	437	174	5.18	19.94

Supplementary Table 10. DAMRatios of alternative Pribnow motifs

Average log10(DAMRatio) of Pribnow in the screen follows the tendency of endogenous alternative Pribnow frequency and Pribnow motif score (up to two mutations in TATAAT allowed). To calculate the average log10(DAMRatio), we used cases having alternative Pribnow motifs in their 1-25 and 1-20 promoter region in the transcription screen. We show that none of the regions are biased towards upstream enrichment of TATAAT motif. *Based on Veronica Llorens-Rico *et al*., see Methods. Canonical Pribnow is TANAAT. Y=yes, N=no.

	**Found in endogenous promoters**		**Pribnow motif score***	**Average log10(DAMRatio) in screen**		
Alternative Pribnow	Count in -15 to -1	Log10 (count)	Pribnow probability	Alternative Pribnow (From 1-25)	Alternative Pribnow (From 1-20)	Canonical Pribnow?
TAAAAT	244	2.387	0.302	1.063	0.933	Y
TATAAT	122	2.086	0.165	1.165	1.042	Y
TAGAAT	83	1.919	0.065	0.886	0.745	Y
TACAAT	77	1.886	0.058	0.991	0.919	Y
TAAGAT	42	1.623	0.027	0.784	0.681	N
TAATAT	27	1.431	0.025	0.959	0.826	N
TATTAT	26	1.415	0.013	0.858	0.709	N
TAACAT	16	1.204	0.017	0.820	0.778	N
TACTAT	15	1.176	0.005	0.746	0.669	N
TATGAT	11	1.041	0.015	0.742	0.669	N
TATCAT	10	1.000	0.009	0.819	0.720	N
TAGTAT	5	0.699	0.005	0.866	0.764	N
TAGCAT	4	0.602	0.004	0.686	0.676	N
TACCAT	3	0.477	0.003	0.696	0.700	N
TAGGAT	3	0.477	0.006	0.686	0.666	N
TACGAT	1	0.000	0.005	0.698	0.676	N
TAAACT	73	1.863	0.047	0.782	0.805	N
TATACT	10	1.000	0.025	0.821	0.816	N
GAAAAT	14	1.146	0.018	0.879	0.763	N
CAAAAT	17	1.230	0.016	0.801	0.688	N
TAAAGT	16	1.204	0.014	0.770	0.701	N
GATAAT	18	1.255	0.010	0.890	0.795	N
TAGACT	4	0.602	0.010	0.670	0.719	N
TACACT	7	0.845	0.009	0.700	0.761	N
CATAAT	21	1.322	0.009	0.900	0.809	N
TATAGT	13	1.114	0.007	0.760	0.717	N
TAAAAC	10	1.000	0.007	0.670	0.692	N

Supplementary Table 11. Odd-ratio differences from the no-constraint case in the transcription screen

Log2 odd-ratio calculated between high- and low-productive promoters for the sequences having the four +1 bases. The numbers represent the differential odd-ratio from all cases. The table is pseudocoloured to highlight the differences.

+1=A

	-37	-36	-35	-34	-33	-32	-31	-30	-29	-28	-27	-26	-25	-24	-23	-22	-21	-20	-19	-18	-17	-16	-15	-14	-13	T	A	T	A	A	T	-6	-5	-4	-3	-2	-1	+1
A	0.0	-0.1	0.0	0.0	0.0	0.0	0.0	0.0	0.0	0.0	0.0	0.1	0.0	0.0	0.0	0.0	0.0	0.0	0.0	0.0	-0.1	0.1	0.0	0.0	0.0							0.0	0.0	0.0	0.0	-0.1	0.1	
C	0.0	0.0	0.0	0.0	0.0	0.0	0.0	-0.1	-0.1	0.0	0.0	0.0	0.0	0.0	0.1	0.0	0.0	0.0	-0.1	0.0	-0.1	-0.3	-0.3	-0.1	-0.1							0.0	0.0	0.1	0.1	0.2	0.2	
G	0.0	0.0	0.0	0.0	0.0	0.0	0.0	0.0	0.0	0.0	0.0	0.0	0.0	0.1	0.0	0.0	0.0	0.0	0.0	0.0	0.0	0.0	-0.3	0.1	0.1							0.0	0.0	0.0	0.1	0.0	-0.6	
T	0.0	0.0	0.0	0.0	0.0	0.0	0.0	0.0	0.0	0.0	0.0	0.0	0.0	0.0	0.0	0.0	0.0	0.0	0.0	0.0	0.0	0.0	0.1	0.0	0.0							0.0	0.0	0.0	-0.1	0.0	0.1	

+1=C

	-37	-36	-35	-34	-33	-32	-31	-30	-29	-28	-27	-26	-25	-24	-23	-22	-21	-20	-19	-18	-17	-16	-15	-14	-13	T	A	T	A	A	T	-6	-5	-4	-3	-2	-1	+1
A	0.0	0.1	0.0	0.0	0.0	0.0	0.0	-0.1	-0.1	0.0	0.0	0.0	0.0	0.0	0.1	0.0	0.1	0.1	0.0	0.0	0.0	-0.1	0.0	0.1	0.0							0.1	0.1	0.0	0.0	0.1	-0.4	
C	0.0	0.0	0.0	0.0	0.0	0.1	0.0	0.0	0.1	0.1	0.0	0.0	0.0	0.0	-0.1	0.0	0.0	-0.1	0.0	0.0	0.0	0.2	0.0	0.1	-0.1							0.0	-0.1	-0.1	-0.2	-0.2	-0.2	
G	0.0	0.0	0.0	0.0	0.0	-0.1	0.0	0.1	-0.1	-0.1	0.0	0.1	0.0	0.0	-0.1	0.0	0.0	0.0	0.0	0.0	0.0	-0.1	0.1	0.0	0.0							0.0	-0.1	0.0	0.0	0.0	-0.1	
T	0.0	0.0	-0.1	0.0	0.0	0.0	0.0	0.0	0.1	0.0	0.0	0.0	0.0	0.0	0.0	0.0	0.0	0.0	0.0	0.0	0.0	0.0	0.0	0.0	0.1							-0.1	0.0	0.0	0.0	0.1	0.5	

+1=G

	-37	-36	-35	-34	-33	-32	-31	-30	-29	-28	-27	-26	-25	-24	-23	-22	-21	-20	-19	-18	-17	-16	-15	-14	-13	T	A	T	A	A	T	-6	-5	-4	-3	-2	-1	+1
A	0.0	0.0	-0.1	0.0	0.0	0.1	0.1	0.1	0.0	0.0	0.1	0.0	0.0	-0.1	0.0	0.0	0.1	0.0	0.0	0.0	-0.1	0.1	0.0	0.0	0.0							0.0	0.0	0.0	0.1	0.0	-0.1	
C	0.0	0.0	0.0	0.0	-0.1	0.0	-0.1	0.0	0.0	0.0	0.0	0.0	0.0	0.1	-0.1	0.0	-0.1	0.0	0.0	-0.1	0.0	-0.3	-0.3	-0.3	-0.1							-0.1	-0.1	-0.1	0.0	-0.1	0.1	
G	0.0	0.0	0.1	0.1	0.0	0.0	0.0	0.0	0.0	0.0	-0.1	0.1	0.0	0.0	0.1	0.0	0.0	0.0	0.1	0.0	-0.2	0.0	-0.2	0.1	0.0							0.0	0.0	-0.1	-0.1	0.1	-0.1	
T	0.0	0.0	0.0	0.0	0.0	0.0	0.0	0.0	0.0	0.0	0.0	0.0	0.0	0.0	0.0	0.0	0.0	0.0	0.0	0.0	0.1	0.0	0.1	0.0	0.0							0.0	0.0	0.1	-0.1	0.0	0.1	

+1=T

	-37	-36	-35	-34	-33	-32	-31	-30	-29	-28	-27	-26	-25	-24	-23	-22	-21	-20	-19	-18	-17	-16	-15	-14	-13	T	A	T	A	A	T	-6	-5	-4	-3	-2	-1	+1
A	0.0	0.0	0.0	0.0	0.0	0.0	0.0	0.0	0.0	0.0	0.0	0.0	0.0	0.1	0.0	0.0	-0.1	0.0	0.0	0.0	0.1	-0.1	0.0	0.0	0.0							0.0	-0.1	0.0	-0.1	0.0	0.2	
C	0.0	0.0	0.0	0.0	0.0	0.0	0.0	0.0	0.0	0.0	0.0	0.0	-0.1	0.0	0.0	0.0	0.1	0.0	0.0	0.1	0.1	0.2	0.2	0.1	0.1							0.1	0.1	0.0	0.0	0.0	-0.1	
G	0.0	0.0	0.0	0.0	0.0	0.1	0.0	0.0	0.0	0.0	0.0	-0.1	0.0	0.0	0.0	0.0	0.0	0.0	0.0	0.0	0.1	0.0	0.2	0.0	0.0							0.0	0.0	0.1	0.0	0.0	0.4	
T	0.0	0.0	0.0	0.0	0.0	0.0	0.0	0.0	0.0	0.0	0.0	0.0	0.0	0.0	0.0	0.0	0.0	0.0	0.0	0.0	-0.1	0.0	-0.1	0.0	0.0							0.0	0.0	0.0	0.1	-0.1	-0.4	

Supplementary Table 12. TSS effect on 4 validation 5'-UTRs and leaderless constructs

The first base of the indicated constructs (UTRs 1,2,7 and 8, Supplementary Table 2 or a leaderless Dam, i.e., they start at the 1st indicated base) were changed to the other 3 bases. Dam protein amounts were determined by Western blot (WB) with an anti-Flag antibody, activity by qPCR (with a genomic "gGATC" or the 4xGATCs of the reporter cassette), and mRNA by RT-qPCR (2 sets of oligos were used; normalized with MPN517 gene). Stdev is the Standard deviation (n=2 for WB, n=3 for qPCR).

a. Effect of the first nucleotide bias in four validation UTR constructs

	Protein		Activity				mRNA			
	WB		qPCR gGATC		qPCR 4xGATC		qPCR dam		qPCR dam 2	
Construct	Average	Stdev	Average	Stdev	Average	Stdev	Average	Stdev	Average	Stdev
SyP32-UTR1-A	5.12	0.53	1.71	0.64	6.81	0.64	6.81	2.54	51.79	15.81
SyP32-UTR1-C	3.58	0.19	4.02	0.75	21.11	0.75	4.63	1.16	21.40	1.28
SyP32-UTR1-G	3.96	1.00	2.93	0.75	9.25	0.75	7.82	0.67	28.93	1.68
SyP32-UTR1-T	3.21	0.70	1.98	0.16	4.73	0.16	6.52	0.50	38.66	4.73
SyP32-UTR2-A	4.18	0.09	1.65	0.27	28.74	0.27	7.43	2.95	30.32	3.62
SyP32-UTR2-C	3.09	0.16	1.39	0.19	2.58	0.19	4.88	1.23	19.14	2.83
SyP32-UTR2-G	4.43	0.82	1.95	0.54	4.67	0.54	7.55	2.29	32.69	4.98
SyP32-UTR2-T	1.78	0.34	0.80	0.29	1.29	0.29	4.54	1.59	15.53	2.66
SyP32-UTR7-A	0.57	0.09	0.42	0.04	0.47	0.04	1.65	0.59	6.0	1.89
SyP32-UTR7-C	0.25	0.07	0.10	0.03	0.01	0.03	1.40	0.40	7.37	0.69
SyP32-UTR7-G	0.45	0.14	0.19	0.06	0.04	0.06	3.07	0.90	16.15	3.26
SyP32-UTR7-T	0.13	0.05	0.07	0.06	0.001	0.06	0.80	0.21	3.17	0.15
SyP32-UTR8-A	0.13	0.07	0.05	0.03	0.002	0.03	0.43	0.05	2.08	0.27
SyP32-UTR8-C	0.04	0.005	0.01	0.01	0.001	0.01	0.35	0.12	2.41	0.15
SyP32-UTR8-G	0.04	0.01	0.04	0.02	0.002	0.02	0.37	0.08	2.65	0.27
SyP32-UTR8-T	0.02	0.002	0.01	0.001	0.001	0.001	0.20	0.09	1.48	0.22

b. Effect of the first nucleotide bias in leaderless constructs

	Protein		Activity				mRNA			
	WB		qPCR gGATC		qPCR 4xGATC		qPCR dam		qPCR dam 2	
Construct	Average	Stdev	Average	Stdev	Average	Stdev	Average	Stdev	Average	Stdev
SyP32-ATG	3.68	0.97473	9.00	2.087	6.41	0.5446	13.03	3.57023	77.68	7.0837
SyP32-CTG	0.31	0.06588	0.00	2E-04	0.01	0.0067	0.59	0.11854	7.89	1.0617
SyP32-GTG	1.17	0.53563	1.85	0.183	1.29	0.12149	8.38	3.2718	82.45	6.07347
SyP32-TTG	0.19	0.1291	0.00	0.001	0.02	0.00325	0.87	0.3111	13.14	2.42451
Ilmp200-ATG	1.19	0.28836	4.12	4.757	10.66	6.59311	4.12	4.75667	10.66	6.59311
Ilmp200-CTG	0.05	0.02917	0.08	0.026	1.02	0.31714	0.08	0.02566	1.02	0.31714
Ilmp200-GTG	0.34	0.00614	0.52	0.053	1.32	0.41171	0.52	0.05314	1.32	0.41171
Ilmp200-TTG	0.03	0.02034	0.32	0.094	3.62	2.7571	0.32	0.09397	3.62	2.7571

Supplementary Table 13. Effect of the translation efficiency on mRNA levels

The dam TSC inside a small (ATC) 5'-UTR was mutated to all other bases. ATG and GTG TSCs have the best translation efficiency. See Supplementary Table 12 legend.

	Protein		Activity				mRNA			
	WB		qPCR gGATC		qPCR 4xGATC		qPCR dam		qPCR dam 2	
Construct	Average	Stdev	Average	Stdev	Average	Stdev	Average	Stdev	Average	Stdev
SyP32-ATC-ATG	5.98	1.30	3.28	0.61	1.87	0.07	108.41	24.76	241.32	89.47
SyP32-ATC-CTG	0.35	0.15	0.03	0.00	0.01	0.004	1.58	0.19	5.84	1.05
SyP32-ATC-GTG	5.88	1.81	0.78	0.18	0.16	0.06	17.68	2.68	43.06	7.41
SyP32-ATC-TTG	4.06	1.57	0.67	0.08	0.08	0.02	18.90	2.46	31.82	36.67

Supplementary Table 14. Endogenous mRNA alternative ATG sites

The number of alternative ATG-containing genes and evaluation of the percentage of genes having an alternative ATG near the TSC. We compared them with randomized sequences as null model.

	# of genes having alternative ATGs in 5'-UTR	# of genes not having alternative ATGs in 5'-UTR	# of genes having ATGs <12nt from the actual TSC	% ATG <12nt
Endogenous genes	265	469	22	8%
Random genes generated with the same length as endogenous gene	308	426	66	21%

Supplementary Table 15. ATG downstream nucleotide effect

Average DAMRatios (log10) when alternative ATG is in-frame (=frame 0) or out-of-frame (=frame 1 or 2). The DAMRatios used here were from Experiment 1 with strong promoter. We divided the sequences according to the distance between the ATG and the TSC (-/+12 nt). Statistically significant cases are highlighted. Stdev is the standard deviation.

a. ATG is located within the -25 to -13 positions of the 5'-UTR

First 5nt	Average log10(DAM Ratio) frame 0	Stdev log10(DAM Ratio) frame 0	Average log10(DAM Ratio) frame 1	Stdev log10(DA MRatio) frame 1	Average log10(DAM Ratio) frame 2	Stdev log10(DA MRatio) frame 2	Difference between in-frame and out-of-frame TSC	*P*-value (t-test)
ATGAA	0.70	0.90	0.62	0.84	0.87	0.91	-0.05	0.52
ATGAC	0.65	0.87	0.44	0.79	0.77	0.88	0.04	0.91
ATGAG	0.79	0.96	0.65	0.92	0.95	0.99	-0.01	0.84
ATGAT	0.73	0.94	0.56	0.87	0.64	0.87	0.13	0.22
ATGCA	0.74	0.99	0.61	0.88	0.88	0.90	-0.01	0.93
ATGCC	0.68	0.85	0.62	0.82	0.63	0.86	0.06	0.64
ATGCG	0.46	0.84	0.64	0.84	0.69	0.81	-0.20	0.09
ATGCT	0.63	0.84	0.78	0.92	0.68	0.83	-0.10	0.47
ATGGA	1.02	0.91	0.92	0.87	0.73	0.93	0.19	0.19
ATGGC	0.51	0.78	0.57	0.83	0.55	0.82	-0.05	0.71
ATGGG	1.09	1.00	0.49	0.86	0.91	0.94	0.40	0.03
ATGGT	0.70	0.92	0.74	0.89	0.76	0.85	-0.05	0.75
ATGTA	0.76	0.81	0.82	0.94	0.70	0.85	0.00	0.95
ATGTC	0.75	0.84	0.67	0.90	0.67	0.81	0.08	0.46
ATGTG	0.68	0.87	0.81	0.88	0.73	0.91	-0.09	0.53
ATGTT	0.57	0.86	0.65	0.91	0.69	0.89	-0.10	0.41

b. ATG is located within the -12 to -3 positions of the 5'-UTR

First 5nt	Average log10(DAM Ratio) frame 0	Stdev log10(DAM Ratio) frame 0	Average log10(DAM Ratio) frame 1	Stdev log10(DA MRatio) frame 1	Average log10(DAM Ratio) frame 2	Stdev log10(DA MRatio) frame 2	Difference between in-frame and out-of-frame TSC	*P*-value (t-test)
ATGAA	0.87	1.03	0.25	0.58	0.11	0.52	0.69	3.32E-16
ATGAC	0.97	1.03	0.23	0.51	0.43	0.72	0.63	1.26E-07
ATGAG	0.75	0.99	0.30	0.73	0.40	0.61	0.40	0.010
ATGAT	0.83	0.96	0.43	0.79	0.32	0.71	0.45	3.87E-06
ATGCA	0.88	1.00	0.45	0.73	0.30	0.64	0.51	9.94E-07
ATGCC	0.70	0.88	0.32	0.64	0.31	0.51	0.39	0.002
ATGCG	0.88	0.92	0.14	0.49	0.27	0.74	0.67	2.18E-07
ATGCT	0.51	0.72	0.40	0.75	0.27	0.68	0.17	0.187
ATGGA	0.69	0.76	0.15	0.45	0.17	0.58	0.53	7.22E-07
ATGGC	0.49	0.80	0.15	0.41	0.31	0.62	0.25	0.057
ATGGG	0.90	0.96	0.15	0.41	0.18	0.45	0.74	3.11E-07
ATGGT	0.66	0.82	0.45	0.82	0.23	0.45	0.32	0.033
ATGTA	0.48	0.78	0.30	0.68	0.97	0.89	-0.16	0.065
ATGTC	1.06	0.97	0.41	0.74	0.58	0.92	0.56	0.0002
ATGTG	0.19	0.50	0.34	0.62	0.38	0.67	-0.18	0.134
ATGTT	0.78	0.87	0.34	0.75	0.46	0.76	0.37	0.017

Supplementary Table 16. Average DAMRatios per base for each position

(a, c) The values represent the average DAMRatios of sequences that have the specific bases at each specific position. (b, d) The average DAMRatios are normalized within the group. The 5'-UTR positions are numbered with respect to the ATG. The table is pseudocoloured to highlight the differences.

a. Average DAMRatios with strong promoter

	5'-UTR position																								
	-25	-24	-23	-22	-21	-20	-19	-18	-17	-16	-15	-14	-13	-12	-11	-10	-9	-8	-7	-6	-5	-4	-3	-2	-1
**Random sequence starting with A**																									
A	1.5	1.6	1.6	1.6	1.6	1.6	1.6	1.6	1.6	1.6	1.6	1.6	1.6	1.6	1.6	1.6	1.6	1.6	1.6	1.6	1.6	1.6	1.7	1.6	1.6
C	NA	1.5	1.4	1.5	1.4	1.5	1.5	1.4	1.5	1.4	1.5	1.5	1.5	1.5	1.5	1.5	1.5	1.5	1.5	1.5	1.5	1.5	1.5	1.5	1.5
G	NA	1.6	1.5	1.5	1.6	1.5	1.6	1.5	1.5	1.5	1.5	1.5	1.4	1.4	1.5	1.4	1.4	1.4	1.4	1.5	1.4	1.5	1.5	1.5	1.3
T	NA	1.5	1.5	1.5	1.5	1.5	1.5	1.5	1.5	1.5	1.5	1.6	1.5	1.6	1.5	1.5	1.5	1.5	1.5	1.6	1.6	1.5	1.5	1.6	1.7
**Random sequence starting with C**																									
A	NA	1.4	1.4	1.3	1.3	1.2	1.2	1.2	1.2	1.2	1.2	1.2	1.2	1.2	1.2	1.2	1.3	1.3	1.3	1.3	1.2	1.3	1.3	1.2	1.2
C	1.2	1	0.9	1.1	1.1	1.2	1.1	1.1	1.1	1.1	1.1	1.1	1.1	1.1	1.2	1.2	1.2	1.2	1.1	1.1	1.1	1.1	1.1	1.2	1.2
G	NA	1	1.2	1.2	1.2	1.3	1.2	1.2	1.2	1.2	1.1	1	1.1	1.1	1.1	1.1	1.1	1.1	1.1	1.1	1.1	1.1	1.1	1.1	1
T	NA	1.2	1.2	1.1	1.1	1.1	1.2	1.2	1.2	1.2	1.2	1.3	1.2	1.2	1.2	1.2	1.2	1.2	1.2	1.2	1.3	1.2	1.1	1.2	1.4
**Random sequence starting with G**																									
A	NA	1.6	1.7	1.6	1.6	1.5	1.5	1.5	1.5	1.5	1.5	1.5	1.5	1.5	1.5	1.5	1.5	1.5	1.6	1.5	1.6	1.6	1.6	1.6	1.5
C	NA	1.3	1.3	1.4	1.4	1.4	1.4	1.4	1.4	1.4	1.4	1.4	1.5	1.4	1.5	1.5	1.5	1.5	1.4	1.4	1.4	1.4	1.4	1.4	1.4
G	1.5	1.4	1.4	1.5	1.5	1.5	1.5	1.5	1.5	1.5	1.4	1.4	1.4	1.4	1.4	1.4	1.3	1.3	1.3	1.4	1.4	1.4	1.4	1.4	1.3
T	NA	1.5	1.5	1.4	1.5	1.4	1.4	1.4	1.4	1.5	1.5	1.5	1.5	1.5	1.5	1.5	1.5	1.5	1.4	1.5	1.5	1.5	1.4	1.5	1.6
**Random sequence starting with T**																									
A	NA	1.1	0.9	0.8	0.8	0.7	0.8	0.8	0.7	0.7	0.7	0.7	0.7	0.7	0.7	0.7	0.7	0.7	0.7	0.7	0.7	0.7	0.7	0.7	0.7
C	NA	0.4	0.4	0.4	0.4	0.5	0.5	0.5	0.6	0.6	0.6	0.6	0.6	0.6	0.6	0.6	0.6	0.6	0.6	0.6	0.6	0.6	0.6	0.6	0.6
G	NA	0.7	0.6	0.6	0.6	0.7	0.8	0.7	0.7	0.6	0.5	0.5	0.5	0.5	0.5	0.6	0.5	0.5	0.5	0.6	0.6	0.6	0.6	0.6	0.5
T	0.6	0.4	0.6	0.6	0.7	0.6	0.5	0.5	0.6	0.6	0.6	0.7	0.7	0.7	0.7	0.7	0.6	0.6	0.6	0.7	0.7	0.6	0.6	0.6	0.7

Average

	-25	-24	-23	-22	-21	-20	-19	-18	-17	-16	-15	-14	-13	-12	-11	-10	-9	-8	-7	-6	-5	-4	-3	-2	-1
A	NA	1.4	1.4	1.3	1.3	1.3	1.3	1.3	1.3	1.3	1.3	1.3	1.3	1.3	1.2	1.2	1.3	1.3	1.3	1.3	1.3	1.3	1.3	1.3	1.2
C	NA	1	1	1.1	1.1	1.1	1.1	1.1	1.1	1.1	1.2	1.2	1.2	1.2	1.2	1.2	1.2	1.2	1.2	1.2	1.2	1.1	1.1	1.2	1.2
G	NA	1.2	1.2	1.2	1.2	1.2	1.3	1.3	1.2	1.2	1.1	1.1	1.1	1.1	1.1	1.1	1.1	1.1	1.1	1.1	1.1	1.1	1.1	1.1	1
T	NA	1.1	1.2	1.1	1.2	1.2	1.1	1.2	1.2	1.2	1.2	1.2	1.2	1.2	1.2	1.2	1.2	1.2	1.2	1.2	1.3	1.2	1.2	1.2	1.3

Standard deviation

	-25	-24	-23	-22	-21	-20	-19	-18	-17	-16	-15	-14	-13	-12	-11	-10	-9	-8	-7	-6	-5	-4	-3	-2	-1
A	NA	0.2	0.3	0.3	0.3	0.3	0.3	0.3	0.3	0.3	0.3	0.3	0.4	0.4	0.4	0.4	0.4	0.4	0.4	0.4	0.4	0.4	0.4	0.4	0.4
C	NA	0.4	0.4	0.4	0.4	0.4	0.4	0.4	0.4	0.3	0.4	0.4	0.4	0.4	0.4	0.4	0.4	0.4	0.3	0.4	0.4	0.4	0.4	0.3	0.4
G	NA	0.3	0.4	0.4	0.4	0.3	0.3	0.3	0.4	0.4	0.4	0.4	0.4	0.4	0.4	0.3	0.3	0.4	0.3	0.4	0.3	0.3	0.3	0.3	0.3
T	NA	0.5	0.4	0.3	0.3	0.4	0.4	0.4	0.4	0.4	0.4	0.4	0.4	0.3	0.3	0.4	0.4	0.4	0.4	0.4	0.3	0.4	0.4	0.4	0.4

b. Normalized DAMRatios within subgroups of strong promoter

	5'-UTR position																								
	-25	-24	-23	-22	-21	-20	-19	-18	-17	-16	-15	-14	-13	-12	-11	-10	-9	-8	-7	-6	-5	-4	-3	-2	-1
**Random sequence starting with A**																									
A	1	1	1.1	1.1	1	1	1	1	1	1	1	1	1	1	1	1	1	1	1.1	1	1	1.1	1.1	1	1
C	NA	1	0.9	1	0.9	1	1	0.9	1	0.9	1	1	1	1	1	1	1	1	1	1	1	1	1	1	1
G	NA	1	1	1	1	1	1	1	1	1	1	1	0.9	0.9	1	0.9	0.9	0.9	0.9	1	0.9	1	1	1	0.9
T	NA	1	1	1	1	1	1	1	1	1	1	1	1	1	1	1	1	1	1	1	1	1	1	1	1.1
**Random sequence starting with C**																									
A	NA	1.2	1.1	1.1	1.1	1	1	1	1	1	1	1	1.1	1	1	1.1	1.1	1.1	1.1	1.1	1	1.1	1.1	1.1	1
C	1	0.8	0.8	0.9	0.9	1	0.9	0.9	0.9	1	1	1	0.9	1	1	1	1	1	1	1	1	0.9	0.9	1	1
G	NA	0.9	1	1	1	1.1	1	1	1	1	0.9	0.9	0.9	0.9	1	0.9	0.9	0.9	0.9	0.9	0.9	0.9	1	0.9	0.9
T	NA	1	1	0.9	1	0.9	1	1	1	1	1	1.1	1	1	1	1	1	1	1	1	1.1	1	1	1	1.2
**Random sequence starting with G**																									
A	NA	1.1	1.1	1.1	1.1	1	1	1	1	1	1	1	1	1	1	1	1	1	1.1	1	1.1	1.1	1.1	1.1	1
C	NA	0.9	0.9	1	0.9	1	1	1	1	1	1	1	1	1	1	1	1	1	1	1	1	0.9	1	1	1
G	1	0.9	0.9	1	1	1	1	1	1	1	1	1	0.9	0.9	1	0.9	0.9	0.9	0.9	0.9	0.9	0.9	0.9	0.9	0.9
T	NA	1	1	0.9	1	1	1	1	1	1	1	1	1	1	1	1	1	1	1	1	1	1	1	1	1.1
**Random sequence starting with T**																									
A	NA	1.7	1.4	1.3	1.2	1.2	1.2	1.2	1.1	1.2	1.1	1.1	1.1	1.1	1.1	1.1	1.1	1.1	1.1	1.1	1.1	1.2	1.2	1.1	1
C	NA	0.6	0.7	0.7	0.7	0.8	0.8	0.8	0.9	0.9	0.9	0.9	0.9	0.9	0.9	0.9	1	0.9	1	0.9	0.9	0.9	0.9	1	1

G	NA	1	0.9	1	1	1.1	1.3	1.2	1.1	1	0.9	0.8	0.9	0.8	0.9	0.9	0.8	0.8	0.8	0.9	0.9	0.9	0.9	0.9	0.8
T	1	0.6	0.9	1	1.1	0.9	0.8	0.8	0.9	0.9	1	1	1	1.1	1.1	1	1	1	1	1	1.1	1	0.9	1	1.2

Average

	-25	-24	-23	-22	-21	-20	-19	-18	-17	-16	-15	-14	-13	-12	-11	-10	-9	-8	-7	-6	-5	-4	-3	-2	-1
A	NA	1.2	1.2	1.1	1.1	1.1	1.1	1.1	1.1	1.1	1.1	1.1	1.1	1	1	1	1	1.1	1.1	1.1	1.1	1.1	1.1	1.1	1
C	NA	0.8	0.8	0.9	0.9	0.9	0.9	0.9	0.9	0.9	1	0.9	1	1	1	1	1	1	1	1	1	0.9	0.9	1	1
G	NA	1	1	1	1	1	1.1	1.1	1	1	0.9	0.9	0.9	0.9	0.9	0.9	0.9	0.9	0.9	0.9	0.9	0.9	1	0.9	0.9
T	NA	0.9	1	0.9	1	1	0.9	0.9	1	1	1	1	1	1	1	1	1	1	1	1	1	1	1	1	1.1

Standard deviation

	-25	-24	-23	-22	-21	-20	-19	-18	-17	-16	-15	-14	-13	-12	-11	-10	-9	-8	-7	-6	-5	-4	-3	-2	-1
A	NA	0.3	0.1	0.1	0.1	0.1	0.1	0.1	0	0.1	0	0	0	0	0	0	0	0	0	0	0	0	0	0	0
C	NA	0.2	0.1	0.1	0.1	0.1	0.1	0.1	0	0	0	0	0	0	0	0	0	0	0	0	0	0	0	0	0
G	NA	0.1	0.1	0	0	0	0.1	0.1	0	0	0	0.1	0	0.1	0	0	0	0.1	0	0	0	0	0	0	0
T	NA	0.2	0	0	0	0	0.1	0.1	0	0	0	0	0	0	0	0	0	0	0	0	0	0	0	0	0

c. Average DAMRatios with weak promoter

	5'-UTR position																								
	-25	-24	-23	-22	-21	-20	-19	-18	-17	-16	-15	-14	-13	-12	-11	-10	-9	-8	-7	-6	-5	-4	-3	-2	-1
**Random sequence starting with A**																									
A	0.4	0.4	0.5	0.5	0.5	0.5	0.4	0.5	0.4	0.4	0.4	0.5	0.5	0.5	0.5	0.5	0.5	0.5	0.5	0.5	0.4	0.5	0.5	0.4	0.4
C	NA	0.3	0.4	0.3	0.3	0.3	0.3	0.3	0.3	0.3	0.3	0.3	0.3	0.3	0.3	0.3	0.3	0.4	0.4	0.4	0.4	0.4	0.4	0.4	0.4
G	NA	0.5	0.5	0.4	0.3	0.3	0.3	0.4	0.4	0.4	0.4	0.4	0.3	0.3	0.3	0.3	0.3	0.3	0.3	0.3	0.4	0.4	0.4	0.3	0.3
T	NA	0.4	0.3	0.4	0.4	0.5	0.5	0.5	0.5	0.4	0.4	0.4	0.4	0.4	0.5	0.4	0.4	0.4	0.4	0.4	0.4	0.4	0.3	0.4	0.6
**Random sequence starting with C**																									
A	NA	0.4	0.4	0.4	0.4	0.4	0.4	0.4	0.4	0.3	0.3	0.4	0.4	0.4	0.4	0.4	0.4	0.4	0.4	0.4	0.4	0.4	0.4	0.4	0.3
C	0.3	0.2	0.3	0.3	0.3	0.2	0.2	0.2	0.2	0.3	0.3	0.3	0.3	0.3	0.3	0.3	0.3	0.3	0.3	0.3	0.3	0.3	0.3	0.3	0.3
G	NA	0.4	0.4	0.3	0.3	0.3	0.3	0.3	0.3	0.3	0.3	0.3	0.3	0.3	0.3	0.3	0.3	0.3	0.3	0.3	0.3	0.3	0.3	0.3	0.2
T	NA	0.3	0.2	0.3	0.3	0.4	0.4	0.4	0.4	0.4	0.4	0.4	0.3	0.3	0.4	0.4	0.4	0.3	0.3	0.3	0.4	0.3	0.3	0.3	0.5
**Random sequence starting with G**																									
A	NA	0.6	0.7	0.7	0.7	0.7	0.6	0.6	0.6	0.6	0.6	0.6	0.6	0.6	0.7	0.7	0.7	0.7	0.7	0.7	0.6	0.7	0.7	0.6	0.6
C	NA	0.5	0.5	0.5	0.5	0.5	0.5	0.5	0.5	0.5	0.5	0.6	0.6	0.6	0.6	0.5	0.5	0.5	0.6	0.6	0.6	0.5	0.5	0.6	0.6
G	0.6	0.7	0.6	0.6	0.5	0.5	0.5	0.6	0.6	0.6	0.6	0.5	0.5	0.5	0.4	0.5	0.5	0.5	0.5	0.5	0.6	0.6	0.6	0.5	0.4
T	NA	0.5	0.5	0.6	0.6	0.7	0.7	0.7	0.6	0.6	0.6	0.6	0.6	0.6	0.6	0.6	0.6	0.6	0.6	0.6	0.6	0.6	0.5	0.6	0.8
**Random sequence starting with T**																									
A	NA	0.6	0.6	0.5	0.5	0.5	0.5	0.5	0.5	0.5	0.5	0.4	0.5	0.5	0.5	0.5	0.5	0.5	0.5	0.5	0.5	0.5	0.6	0.4	0.4
C	NA	0.2	0.4	0.3	0.3	0.3	0.3	0.3	0.3	0.3	0.4	0.4	0.4	0.4	0.3	0.3	0.3	0.4	0.4	0.4	0.4	0.4	0.4	0.4	0.4
G	NA	0.3	0.4	0.4	0.4	0.4	0.4	0.4	0.4	0.4	0.4	0.4	0.4	0.3	0.3	0.3	0.4	0.3	0.3	0.3	0.4	0.4	0.4	0.4	0.3
T	0.4	0.2	0.3	0.4	0.4	0.5	0.5	0.5	0.5	0.4	0.4	0.5	0.4	0.5	0.5	0.5	0.5	0.5	0.4	0.5	0.4	0.4	0.3	0.4	0.6

Average

	-25	-24	-23	-22	-21	-20	-19	-18	-17	-16	-15	-14	-13	-12	-11	-10	-9	-8	-7	-6	-5	-4	-3	-2	-1
A	NA	0.5	0.5	0.5	0.5	0.5	0.5	0.5	0.5	0.5	0.5	0.5	0.5	0.5	0.5	0.5	0.5	0.5	0.5	0.5	0.5	0.5	0.6	0.5	0.4
C	NA	0.3	0.4	0.3	0.3	0.3	0.3	0.3	0.3	0.4	0.4	0.4	0.4	0.4	0.4	0.4	0.4	0.4	0.4	0.4	0.4	0.4	0.4	0.4	0.4
G	NA	0.5	0.4	0.4	0.4	0.4	0.4	0.4	0.4	0.4	0.4	0.4	0.4	0.4	0.3	0.3	0.4	0.3	0.4	0.4	0.4	0.4	0.4	0.4	0.3
T	NA	0.4	0.3	0.4	0.5	0.5	0.5	0.5	0.5	0.5	0.5	0.5	0.5	0.5	0.5	0.5	0.5	0.5	0.4	0.5	0.5	0.4	0.4	0.5	0.6

Standard deviation

	-25	-24	-23	-22	-21	-20	-19	-18	-17	-16	-15	-14	-13	-12	-11	-10	-9	-8	-7	-6	-5	-4	-3	-2	-1
A	NA	0.1	0.1	0.1	0.1	0.1	0.1	0.1	0.1	0.1	0.1	0.1	0.1	0.1	0.1	0.1	0.1	0.1	0.1	0.1	0.1	0.1	0.1	0.1	0.1
C	NA	0.1	0.1	0.1	0.1	0.1	0.1	0.1	0.1	0.1	0.1	0.1	0.1	0.1	0.1	0.1	0.1	0.1	0.1	0.1	0.1	0.1	0.1	0.1	0.1
G	NA	0.2	0.1	0.1	0.1	0.1	0.1	0.1	0.1	0.1	0.1	0.1	0.1	0.1	0.1	0.1	0.1	0.1	0.1	0.1	0.1	0.1	0.1	0.1	0.1
T	NA	0.1	0.1	0.1	0.1	0.1	0.1	0.1	0.1	0.1	0.1	0.1	0.1	0.1	0.1	0.1	0.1	0.1	0.1	0.1	0.1	0.1	0.1	0.1	0.1

d. Normalized DAMRatios within subgroups of weak promoter

	5'-UTR position																								
	-25	-24	-23	-22	-21	-20	-19	-18	-17	-16	-15	-14	-13	-12	-11	-10	-9	-8	-7	-6	-5	-4	-3	-2	-1
**Random sequence starting with A**																									
A	1	1	1.2	1.2	1.2	1.1	1.1	1.1	1.1	1.1	1.1	1.1	1.2	1.2	1.2	1.2	1.2	1.2	1.1	1.1	1.1	1.2	1.3	1.1	1
C	NA	0.7	0.9	0.8	0.7	0.7	0.7	0.7	0.8	0.8	0.8	0.8	0.8	0.8	0.8	0.8	0.8	0.9	0.9	0.9	0.9	0.9	0.9	1	0.9
G	NA	1.3	1.1	0.9	0.9	0.8	0.8	0.9	0.9	1	1	0.9	0.8	0.8	0.7	0.7	0.8	0.8	0.8	0.9	0.9	0.9	1	0.8	0.7

T	NA	0.9	0.8	0.9	1	1.2	1.2	1.1	1.1	1.1	1.1	1.1	1.1	1.1	1.1	1.1	1.1	1.1	1	1	1	1	0.8	1.1	1.4

Random sequence starting with C

A	NA	1.2	1.2	1.2	1.2	1.2	1.1	1.1	1.1	1	1	1.1	1.1	1.2	1.2	1.2	1.1	1.2	1.2	1.2	1.1	1.2	1.3	1.1	1
C	1	0.7	0.9	0.9	0.7	0.7	0.7	0.7	0.7	0.9	0.9	0.8	0.8	0.8	0.9	0.9	0.8	0.9	0.9	0.9	0.9	0.8	0.8	1	0.9
G	NA	1.2	1.1	0.9	0.9	0.9	0.9	0.9	1	1	1	1	0.9	0.8	0.8	0.8	0.9	0.8	0.8	0.8	0.9	1	1	0.9	0.7
T	NA	0.8	0.7	0.9	1	1.1	1.2	1.2	1.1	1.1	1.1	1.1	1	1	1.1	1.1	1.1	1	1	1	1.1	0.9	0.8	1	1.4

Random sequence starting with G

A	NA	1.1	1.2	1.2	1.1	1.1	1.1	1.1	1.1	1	1	1	1.1	1.1	1.1	1.1	1.1	1.1	1.1	1.1	1.1	1.1	1.2	1.1	1
C	NA	0.8	0.8	0.8	0.8	0.8	0.8	0.8	0.8	0.9	0.9	1	0.9	0.9	0.9	0.9	0.9	0.9	0.9	0.9	0.9	0.9	0.9	1	1
G	1	1.2	0.9	1	0.9	0.8	0.9	1	1	1	0.9	0.9	0.8	0.8	0.7	0.8	0.8	0.8	0.8	0.8	0.9	0.9	1	0.9	0.7
T	NA	0.9	0.9	1	1.1	1.1	1.1	1.1	1.1	1.1	1.1	1.1	1	1.1	1.1	1.1	1.1	1.1	1	1	1	1	0.8	1	1.3

Random sequence starting with T

A	NA	1.5	1.3	1.2	1.3	1.2	1.1	1.1	1.1	1.1	1.1	1.1	1.1	1.1	1.2	1.2	1.2	1.1	1.1	1.1	1.1	1.2	1.3	1.1	1
C	NA	0.6	0.9	0.8	0.7	0.7	0.7	0.7	0.7	0.8	0.8	0.9	0.9	0.9	0.8	0.8	0.8	0.9	0.9	0.9	0.9	0.9	0.9	1	0.9
G	NA	0.7	0.9	0.9	0.8	0.8	0.9	1	1	1	1	0.9	0.8	0.8	0.7	0.8	0.8	0.7	0.8	0.8	0.9	1	1	0.9	0.7
T	1	0.6	0.8	1	1	1.1	1.2	1.1	1.1	1.1	1.1	1.1	1	1.1	1.1	1.1	1.1	1.1	1	1.1	1.1	0.9	0.8	1	1.4

Average

	-25	-24	-23	-22	-21	-20	-19	-18	-17	-16	-15	-14	-13	-12	-11	-10	-9	-8	-7	-6	-5	-4	-3	-2	-1
A	NA	1.2	1.2	1.2	1.2	1.2	1.1	1.1	1.1	1.1	1	1.1	1.1	1.2	1.2	1.2	1.1	1.2	1.1	1.1	1.1	1.2	1.3	1.1	1
C	NA	0.7	0.9	0.8	0.7	0.7	0.7	0.8	0.8	0.8	0.9	0.9	0.9	0.8	0.9	0.8	0.8	0.9	0.9	0.9	0.9	0.9	0.9	1	0.9
G	NA	1.1	1	0.9	0.9	0.8	0.9	0.9	1	1	1	0.9	0.9	0.8	0.7	0.8	0.8	0.8	0.8	0.8	0.9	1	1	0.9	0.7
T	NA	0.8	0.8	0.9	1	1.1	1.2	1.1	1.1	1.1	1.1	1.1	1	1.1	1.1	1.1	1.1	1	1	1	1	1	0.8	1	1.4

Standard deviation

	-25	-24	-23	-22	-21	-20	-19	-18	-17	-16	-15	-14	-13	-12	-11	-10	-9	-8	-7	-6	-5	-4	-3	-2	-1
A	NA	0.2	0	0	0.1	0	0	0	0	0	0	0	0	0.1	0	0	0	0	0	0	0	0	0	0	0
C	NA	0.1	0	0	0	0	0	0	0	0	0	0.1	0	0.1	0	0	0	0	0	0	0	0	0	0	0
G	NA	0.2	0.1	0.1	0	0	0	0	0	0	0	0.1	0	0	0	0	0	0	0	0	0	0	0	0	0
T	NA	0.1	0.1	0	0	0	0	0	0	0	0	0	0	0	0	0	0	0	0	0	0	0	0	0	0.1

Supplementary Table 17. Shine-Dalgarno sequence effect on the translation screens

The sequences are divided into two groups: with or without Shine-Dalgarno (SD)-like sequence (GAGG, GGAG, GAAG, and AGGA) scanning from -20 bps upstream the ATG. Then sequences are also binned according to their 5´-UTR folding energies (0-55 nt). We only analysed those 5'-UTR sequences having no alternative ATG.

Strong promoter

	SD			No SD				Folding energy	
**Bin**	**Average DAMRatio (log10)**	**Stdev**	**# of sequences**	**Average DAMRatio (log10)**	**Stdev**	**# of sequences**	**T-test**	**Average ΔG (0-55)**	**Stdev ΔG**
1	0.68	0.89	2198	0.57	0.85	10602	2.47E-08	-11.50	0.59
2	1.21	0.93	1632	1.10	0.90	11014	7.92E-06	-8.79	1.12
3	1.44	0.90	1444	1.30	0.90	11160	6.01E-08	-7.36	1.32
4	1.58	0.88	1209	1.42	0.92	11404	1.56E-08	-6.10	1.44
5	1.62	0.89	900	1.49	0.92	11757	5.62E-05	-4.54	1.50
No bin	1.21	0.97	7383	1.19	0.96	55937	1.06E-01		

Weak promoter

	SD			No SD				Folding energy	
**Bin**	**Average DAMRatio (log10)**	**Stdev**	**# of sequences**	**Average DAMRatio (log10)**	**Stdev**	**# of sequences**	**T-test**	**Average ΔG (0-55)**	**Stdev ΔG**
1	0.13	0.43	1661	0.11	0.41	8058	7.87E-02	-11.52	0.11
2	0.36	0.55	1250	0.31	0.52	8347	1.09E-03	-8.95	0.31
3	0.55	0.60	1145	0.46	0.59	8470	2.02E-06	-7.56	0.47
4	0.65	0.62	1022	0.58	0.63	8781	7.66E-04	-6.29	0.59
5	0.79	0.65	844	0.72	0.70	9220	3.45E-03	-4.69	0.72
No bin	0.44	0.61	5922	0.45	0.62	42876	8.47E-01		

Supplementary Table 18. Overall base preference of N7 and N8 dam mRNAs

The numbers represent the total number of sequences that have the indicated bases at each position in RNA-seq. For N7 and N8 mRNAs (starting at natural +1 and +2, respectively), sequences are aligned to the experimentally determined TSS (position +1). The numbering on top is based on the randomized positions (N25) after the promoter. Sequences having an identical TSS in both RNA-seq approaches were used.

a. Common TSSs from RNA-seq experiments

N7	1	2	3	4	5	6	7	8	9	10	11	12	13	14	15	16	17	18	19	20	21	22	23	24	25	26
A	0	69	2258	1558	1607	1470	1319	1353	1383	1470	1393	1392	1497	1398	1440	1361	1269	1478	1617	1762	1364	1351	1393	1308	1264	1605
C	0	4188	849	976	952	1055	987	951	946	924	1059	1029	1032	1067	1028	1161	1158	1341	1156	1030	1127	1063	1106	958	986	947
G	0	176	1153	958	1045	1048	1051	931	1010	972	919	845	707	767	753	795	709	605	672	710	900	999	916	977	1137	1105
T	4637	204	377	1145	1033	1064	1280	1402	1298	1271	1266	1371	1401	1405	1416	1320	1501	1213	1192	1135	1246	1224	1222	1394	1250	980

N8	1	2	3	4	5	6	7	8	9	10	11	12	13	14	15	16	17	18	19	20	21	22	23	24	25
A	8148	1707	3133	3182	3242	3172	2958	2921	2999	2924	2833	2961	3260	2996	2975	3111	2873	3414	3872	2811	2768	2953	2636	2830	3550
C	9	2906	1971	2435	2073	1941	2070	1989	2151	2231	2231	2301	2290	2161	2209	2268	3105	2610	2196	2380	2429	2339	2107	2192	2091
G	1578	1546	2722	1954	2023	2338	2260	2061	1990	1975	1841	1659	1348	1513	1720	1618	1398	1325	1494	1979	2096	1909	2065	2280	2202
T	128	3704	2037	2292	2525	2412	2575	2892	2723	2733	2958	2942	2965	3193	2959	2866	2487	2514	2301	2693	2570	2662	3055	2561	2020

b. TSSs from the 1st RNA-seq approach

N7	1	2	3	4	5	6	7	8	9	10	11	12	13	14	15	16	17	18	19	20	21	22	23	24	25	26
A	0	2289	6832	5314	5453	4550	4438	4767	4798	4971	4745	4858	5247	4661	4907	4565	4187	5114	5500	5896	4656	4440	4756	4230	4302	5445
C	0	8425	2893	3258	2922	3224	3460	3046	3065	3019	3458	3434	3251	3251	3268	3706	3839	4186	3774	3335	3613	3635	3566	3379	3352	3272
G	0	3058	3131	2822	3169	3845	3088	2887	3351	3131	2901	2669	2478	2758	2445	2552	2339	1981	2282	2220	2946	3168	3002	3218	3640	3474
T	15327	1555	2471	3933	3783	3708	4341	4627	4113	4206	4223	4366	4351	4657	4707	4504	4962	4046	3771	3876	4112	4084	4003	4500	4033	3136

N8	1	2	3	4	5	6	7	8	9	10	11	12	13	14	15	16	17	18	19	20	21	22	23	24	25
A	14897	4221	6558	6727	6797	6700	6126	6201	6336	6039	6082	6288	6950	6221	6354	6623	6048	7148	8076	5991	5886	6207	5557	5950	7389
C	207	5885	4222	4697	4224	3958	4402	4309	4470	4670	4676	4728	4743	4567	4669	4697	6453	5349	4563	4919	5042	4860	4542	4506	4320
G	4302	3587	4723	3818	3858	4627	4536	4141	4147	4158	3735	3414	2736	3149	3428	3317	2845	2750	3134	3977	4271	4041	4163	4747	4597
T	1185	6898	5088	5349	5712	5306	5527	5940	5638	5724	6098	6161	6162	6654	6140	5954	5245	5344	4818	5704	5392	5483	6329	5388	4285

c. TSSs from the 2nd RNA-seq approach

N7	1	2	3	4	5	6	7	8	9	10	11	12	13	14	15	16	17	18	19	20	21	22	23	24	25	26
A	0	454	3669	2784	2831	2648	2344	2445	2526	2588	2439	2500	2606	2533	2535	2425	2367	2655	2857	3159	2471	2416	2495	2353	2280	2848
C	0	6250	1588	1755	1731	1781	1742	1691	1612	1604	1869	1781	1793	1857	1812	1967	1977	2342	2023	1799	1982	1840	1936	1718	1770	1686
G	0	513	2097	1623	1772	1743	1833	1579	1729	1686	1604	1468	1317	1328	1268	1376	1213	1056	1137	1233	1537	1719	1586	1678	1937	1875
T	8195	978	841	2033	1861	2023	2276	2480	2328	2317	2283	2446	2479	2477	2580	2427	2638	2142	2178	2004	2205	2220	2178	2446	2208	1786

N8	1	2	3	4	5	6	7	8	9	10	11	12	13	14	15	16	17	18	19	20	21	22	23	24	25
A	13334	4418	6211	5807	5692	5697	5604	5570	5589	5499	5443	5678	5816	5566	5392	5517	5690	6440	7187	5295	5180	5441	4891	5205	6406
C	201	4999	3516	4176	3853	3730	3645	3549	3844	4007	3991	3972	4027	3880	4075	4179	5382	4631	3910	4327	4352	4203	3957	3975	3804
G	3926	2817	4465	3754	3983	3997	3988	3858	3621	3523	3273	3042	2715	2779	3033	2898	2447	2391	2607	3512	3779	3466	3672	3992	3997
T	594	5821	3863	4318	4527	4631	4818	5078	5001	5026	5348	5363	5497	5830	5555	5461	4536	4593	4351	4921	4744	4945	5535	4883	3848

Supplementary Table 19. Average RNA copy number in function of base identity at each position

RNA copy number from two RNA-seq experiments were combined and normalized by DNA copy number from the 'uncut' library (Combined RNA TPM / log10(DNACopy)) (Arbitrary units) in the two translation screens. TPM is 'transcripts per million' (number of RNA-seq reads for certain constructs / total RNA-seq reads * 10^6, see Methods). The 5'-UTR positions are numbered with respect to the ATG.

a. Strong promoter

	5'-UTR position																								
	-25	-24	-23	-22	-21	-20	-19	-18	-17	-16	-15	-14	-13	-12	-11	-10	-9	-8	-7	-6	-5	-4	-3	-2	-1
A	0.91	0.86	0.86	0.86	0.87	0.86	0.86	0.86	0.86	0.86	0.86	0.86	0.86	0.86	0.86	0.86	0.86	0.86	0.86	0.86	0.86	0.86	0.87	0.86	0.86
C	0.86	0.87	0.85	0.87	0.86	0.86	0.86	0.86	0.86	0.86	0.87	0.86	0.87	0.86	0.86	0.87	0.87	0.87	0.86	0.86	0.86	0.86	0.86	0.86	0.86
G	0.83	0.86	0.90	0.88	0.87	0.87	0.87	0.87	0.87	0.87	0.86	0.86	0.86	0.87	0.87	0.87	0.87	0.86	0.86	0.86	0.86	0.86	0.86	0.86	0.86
T	0.80	0.86	0.84	0.84	0.85	0.85	0.85	0.85	0.85	0.86	0.86	0.86	0.86	0.86	0.86	0.86	0.86	0.86	0.86	0.86	0.86	0.86	0.86	0.86	0.87

b. Weak promoter

	-25	-24	-23	-22	-21	-20	-19	-18	-17	-16	-15	-14	-13	-12	-11	-10	-9	-8	-7	-6	-5	-4	-3	-2	-1
A	0.85	0.91	0.90	0.91	0.91	0.91	0.91	0.91	0.91	0.90	0.91	0.91	0.91	0.91	0.91	0.90	0.91	0.91	0.91	0.91	0.91	0.91	0.92	0.91	0.91
C	0.95	0.88	0.93	0.91	0.91	0.91	0.91	0.91	0.91	0.91	0.91	0.91	0.91	0.91	0.90	0.91	0.91	0.91	0.91	0.91	0.91	0.91	0.91	0.91	0.91
G	0.89	0.97	0.93	0.92	0.92	0.91	0.91	0.91	0.91	0.92	0.92	0.91	0.92	0.91	0.91	0.92	0.91	0.91	0.91	0.91	0.91	0.91	0.91	0.91	0.91
T	0.93	0.85	0.87	0.89	0.90	0.91	0.91	0.91	0.91	0.90	0.90	0.91	0.90	0.90	0.91	0.91	0.91	0.90	0.90	0.90	0.91	0.90	0.90	0.91	0.91

Supplementary Table 20. RNA-seq counts for validation 5'-UTRs

Dam-specific RNA-seq of eight 5'-UTRs from the translation screen. Numbers are read counts, not barcode counts. N7 and N8 indicate the number of reads starting from the theoretical TSS (+1) and +2, respectively. As we found a possible contamination from the 5´-UTR pool of UTR1-8 in RNA-seq approach 1, we do show the read counts only for the second approach. See also Supplementary Table 19 legend.

	UTR8 Pool			ELM-seq Exp1			ELM-seq Exp2			RNA-seq Exp2								
**ID**	**N7 RNA counts**	**N8 RNA counts**	**Sum**	**uncut**	**DpnI**	**MboI**	**uncut**	**DpnI**	**MboI**	**N7 3**	**N8 3**	**Sum**	**Combined DAMRatio**	**DNACopy TPM 1**	**DNACopy TPM 2**	**Combined DNA CopyTPM**	**RNACopy TPM 2**	**Normalized RNA CopyTPM**
UTR1	5881	559121	620240	48	0	149	42	17	255	34	1049	1255	2.62	4.28E+03	3.90E+03	1.20E+01	1.23E+04	1.14E+04
UTR2	2485	240304	272439	35	4	114	26	2	70	108	2106	2399	2.54	3.12E+03	2.41E+03	1.14E+01	2.35E+04	2.22E+04
UTR3	357157	8479	393825	62	7	145	64	12	162	4750	324	5263	2.14	5.52E+03	5.94E+03	1.25E+01	5.15E+04	4.70E+04
UTR4	10098	147995	167262	67	19	249	1	1	17	95	3421	3791	2.27	5.97E+03	9.29E+01	9.54E+00	3.71E+04	3.79E+04
UTR5	163	64	102933	334	87	684	157	26	320	0	0	850	1.94	2.98E+04	1.46E+04	1.43E+01	8.33E+03	7.20E+03
UTR6	99476	1530	153984	209	152	247	102	55	119	167	0	421	1.23	1.86E+04	9.47E+03	1.37E+01	4.12E+03	3.63E+03
UTR7	104940	1577	111376	68	138	17	24	18	102	237	0	237	0.88	6.06E+03	2.23E+03	1.18E+01	2.32E+03	2.16E+03
UTR8	263	583	4415	187	364	23	38	11	251	0	0	0	1.03	1.67E+04	3.53E+03	1.29E+01	0.00E+00	0.00E+00

Supplementary Note 1. Library cloning and sequencing

Library cloning. The number of possible DNA sequences was 4^{32} (10^{19}) for the promoter and 4^{25} (10^{15}) in the case of the 5´-UTR libraries. In order to obtain sufficient variability in the random sequences, we followed the strategy detailed below. The random part of the sequence was ordered as any base or "N" in the oligonucleotides to the manufacturer (maintaining the normal GC content of *M. pneumoniae*, i.e., ca. 40%). In the case of the transcription screen, the oligo with the random sequence was used to amplify *dam* (Supplementary Fig. 11). In this way, a different sequence was introduced in each PCR cycle. In the case of the translation screen the random sequence was introduced as a linker, in which the second strand was made by primer extension (Supplementary Fig. 11).

From the controls of *E. coli* transformation efficiency (about 10^9 cells per µg DNA) we estimated approximately 1 million colonies were obtained per construct. This is consistent with the number of different sequences that were obtained in the sequencing output. Nevertheless, not all the sequences were used after applying the filter in the DAMRatio calculation (see Methods).

Library sequencing. The setup of the DNA-seq and RNA-seq deep sequencing strategies are shown schematically in Supplementary Fig. 12. We used customized oligos with the Illumina flow cell binding (P5 and P7) and sequencing (SEQ) regions. In the case of DNA-seq, there was only one step (PCR amplification from genomic DNA, digested with either DpnI or MboI) needed to obtain a library for sequencing the Dam cassette. In the case of RNA-seq however, we tested two strategies to enable sequencing of the *dam* mRNA. In one case, the "specificity" was achieved by *dam* specific RT, while in the other, it was introduced in the last step (by PCR from bulk cDNA; see Supplementary Note 3). It should be noted that a random sequence of six bases was introduced in the linker used for ligation of the ssDNA (or cDNA). The reason is that, like this, individual RNA species are also coded. In this way, we can remove (bioinformatically) the ones that arise from duplications of original sequences in the final PCR (that PCR needed to obtain the final libraries).

RNA-seq was only possible in the 5´-UTR screens, as the "barcode" (i.e., the N25 randomized sequence) is in the RNA itself.

Supplementary Note 2. DAMRatio distribution fitting

With a mixture of Gaussian fitting, we obtained i- or tri-modal distributions of DAMRatios depending on the study. To understand this, we simulated our experimental scheme with some basic assumptions: (1) Expression from random sequences generates a Gaussian distribution of DAM expression. (2) There is more than one clone that has same sequence in the population. It is realistic that we might have more than one isogenic clone in the *M. pneumoniae* population. During the library preparation, we cloned random libraries into *E. coli* before extracting the DNA for *M. pneumoniae* transformation. Since we made two passages before extracting the DNA and amplifying it by PCR in the following step, we considered the number of average isogenic clones in the population is more than one (for the simulation we set this as ten). (3) A DNA methylation event in a single cell is considered as a random process - its probability is proportionally dependent on the concentration of Dam enzyme. Thus, we assume that methylation at one site does not affect the methylation of other sites. To model the methylation process, we used a sigmoid probability distribution of Dam amount along the cell cycle. As Dam expression increases, the probability of having DNA methylation also increases. (4) Similar as for Dam, restriction enzyme cutting was also modeled as a random process. In this way, sometimes DNA is not cut. We used a 95% cutting enzyme efficiency for the simulation. (5) PCR can also randomly amplify specific constructs. We set the amplification probability as 95% for each cycle, and ran 10 cycles.

Using these schemes, we obtained the same distribution in the simulation results (Supplementary Fig. 13). The enrichment of high- and low-productive clones can be explained by the partial methylation of the 4xGATC sites. If the GATC sites of a sequence are not fully methylated or un-methylated, then both DpnI and MboI are capable of cutting the reporter site. This consequently leads to fragments that cannot be amplified during the PCR step. Thus, we hypothesized that the enrichment of both types of (highly and lowly expressed) sequences is governed by the number of GATC sites. As expected, increasing the number of GATC sites resulted in an enrichment of both highly expressed promoters and lowly expressed ones (see Supplementary Fig. 13).

GATC sites could be hemimethylated if the cells have just replicated their DNA. DNA. Both DpnI and MboI are not capable of cutting the hemimethylated DNA (less than 2%)[1-5]. To test the possible impact of hemimethylation we run a simulation considering separated methylation events on each strand. Supplementary Fig. 14 shows the probability of being methylated or hemimethylated depending on the expression level of Dam. In summary, hemimethylation does not change the conclusions of the simulation described above. The only effect is that hemimethylated DNA can increase the number of reads coming from intermediate level expression of Dam (Supplementary Fig. 15).

Supplementary Note 3. RNA-seq validation

The two RNA-seq approaches (see Supplementary Note 1) show a reasonable correlation (RNA1 and RNA2 in Supplementary Fig. 16).

The correlation of RNA-seq with Dam activity (DAMRatio) was significant, but not so good (Supplementary Fig. 16). This was expected as the determinants of RNA expression and translation are not the same. However, it is worth noting that when we look at the validation constructs (12 promoters and eight 5'-UTRs; RT-qPCR data in Supplementary Table 5), the correlation of DAMRatio with mRNA levels is r=0.44/0.25 (two different qPCR oligos for *dam*). RNA shows also a good correlation with Dam protein levels as determined by LC-MSMS (r=0.73/0.83).

We also performed another control to test the RNA-seq protocol. Briefly, equal amounts of the total RNA of the eight individually expressed 5´-UTR validation constructs (UTR1-8 in Supplementary Table 2) were pooled and the two RNA-seq approaches were applied. The amount of RNA produced by these constructs was determined by RT-qPCR. As it can be seen in Supplementary Table 20, the correlation of the *dam* mRNA counts found in the original libraries (corrected by the genomic DNA counts from the uncut sequencing of the reporter cassette) and the pooled 5´-UTRs (no need to correct, as they were normalized when pooling the same amount of total RNA of each 5´-UTR) is high (r=0.41). As a matter of fact, there is also a correlation of the mRNA of the individual clones determined by qPCR with that obtained by RNA-seq in the pools (r = 0.84 and 0.68 for the 2 sets of *dam* oligos), indicating that our customized mRNA-specific RNA-seq protocol reflects the original RNA present in the cell.

Regarding sequence determinants of the mRNA levels, the base preferences per position in the random sequence can be found in Supplementary Table 19. The only remarkable feature is an enrichment of ANG sequences (AYN in the weak promoter) at the beginning of the RNA (it has to be considered that the strong promoter usually starts at the first base of the randomized sequence while the weak promoter does so at one position before, that is, an A, thereby displacing this motif by one base). This could be related to the preference of the RNA polymerase for the first incorporated base. Nevertheless, this is not reflected in higher protein expression according to DAMRatio analysis (see base preferences in Fig. 3), indicating that there are other factors having greater influence over the *dam* translation.

Supplementary Note 4. Alternative ATG effect on the translation

A confounding factor in the analysis of the 5'-UTR sequences is the possible introduction of alternative TSCs in the random sequence. Alternative TSCs could hamper the translation of Dam by competing for access to the ribosome initiation complex. The theoretical probability of having alternative ATG sites in the library is significant (35.9%), and in fact, we found that 34.3% and 32% of the sequences have at least one ATG in the 5'-UTR for the strong and weak promoter screen, respectively. As expected, these alternative TSCs systematically reduced Dam activity (Supplementary Fig. 8a). The greatest reduction was seen in the cases where the TSCs were out-of-frame and closely located to the real one (≤12 nt) (Supplementary Fig. 8b). In fact, the degree to which an alternative TISs affects the translation of the Dam depends on the distance between TISs. In agreement with this, we found that endogenous *M. pneumoniae* genes tend to be scarce in alternative ATGs near their TSCs ($P = 1.43 \times 10^{-6}$, Fisher's exact test; Supplementary Table 14). We further found that the nucleotides directly around the alternative TSCs also play a role (Supplementary Table 15 and Supplementary Fig. 8c,d).

Supplementary References

1. Davis, T. B., Morgan, R. D., and Robinson, D. P. DpnI cleaves Hemimethylated DNA. *In Human Genome II, Official Program and Abstracts. San Deigo, CA.*, p. 26. (1990).
2. Hermann, A. & Jeltsch, A. Methylation sensitivity of restriction enzymes interacting with GATC sites. *Biotechniques* **34**, 924-926, 928, 930 (2003).
3. Nelson, M., Christ, C. & Schildkraut, I. Alteration of apparent restriction endonuclease recognition specificities by DNA methylases. *Nucleic Acids Res* **12**, 5165-5173 (1984).
4. Ono, A. & Ueda, T. Synthesis of decadeoxyribonucleotides containing N6-methyladenine, N4-methylcytosine, and 5-methylcytosine: recognition and cleavage by restriction endonucleases (nucleosides and nucleotides part 74). *Nucleic Acids Res* **15**, 219-232 (1987).
5. Streeck, R. E. Single-strand and double-strand cleavage at half-modified and fully modified recognition sites for the restriction nucleases Sau3a and Taqi. *Gene* **12**, 267-275 (1980).
6. Llorens-Rico, V., Lluch-Senar, M. & Serrano, L. Distinguishing between productive and abortive promoters using a random forest classifier in Mycoplasma pneumoniae. *Nucleic Acids Res* **43**, 3442-3453, doi:10.1093/nar/gkv170 (2015).
